# Metabolomic Investigation of Myelodysplastic Syndromes, Multiple Myeloma, and Homozygous β-Thalassemia

**DOI:** 10.3390/cells14221788

**Published:** 2025-11-14

**Authors:** Elena Chatzikalil, Konstantinos Bistas, Vasiliki Kymioni, Panagiotis T. Diamantopoulos, Elena E. Solomou

**Affiliations:** 1 First Department of Pediatrics, National and Kapodistrian University of Athens Medical School, 11527 Athens, Greece; elenachatz@med.uoa.gr (E.C.); bistaskon@med.uoa.gr (K.B.); vmariakymioni@gmail.com (V.K.); 2 Aghia Sofia Children’s Hospital ERN-PeadCan Center, 11527 Athens, Greece; 3 Department of Internal Medicine, National and Kapodistrian University of Athens Medical School, 11527 Athens, Greece; pandiamantopoulos@gmail.com; 4 Department of Internal Medicine, University of Patras Medical School, 26500 Rion, Greece

**Keywords:** metabolomics, metabolites, chronic anemia, cellular aging, myelodysplasia, thalassemia

## Abstract

Chronic anemia is commonly diagnosed in older adults and serves an important indicator of both reactive and clonal conditions. Many underlying diseases, such as myelodysplastic syndromes and multiple myeloma, are more prevalent amongst the elderly, while novel therapeutic approaches have transformed pediatric disorders of poor prognosis, such as beta-thalassemia, to a chronic disease of older adults. Thus, the increasing prevalence of chronic anemia in older ages is largely attributed to more frequent diagnostic and therapeutic evaluations and demographic changes. The etiology of anemia in adults is complex, ranging from genetic mutations to bone marrow failure syndromes, chronic kidney disease, nutritional deficiencies, and inflammatory processes, while in some cases no clear etiology is found. For this reason, extensive research is ongoing to introduce novel therapeutic targets and improve quality of life. Management of anemia in adults depends on severity and especially on the underlying conditions of each patient. Metabolic pathway analyses have revealed alterations in various pathways, including glycolysis, pyruvate, propanoate, glycerophospholipid, galactose, fatty acid, starch, and sucrose metabolism along with fatty acid elongation in mitochondria, glycerolipid, glyoxylate, and dicarboxylate metabolism in adult patients with chronic anemia compared to healthy individuals, which may serve as potential new therapeutic targets. In this review, we aim to (i) summarize current evidence regarding metabolic disturbances in diseases of age-related hematopoietic dysregulation, being represented by multiple myeloma and myelodysplastic syndromes, and in β-thalassemia, a disease model of accelerating cellular aging; and (ii) describe novel therapeutic metabolic-targeted options for the aforementioned diseases, focusing on the need for continuous research in this field.

## 1. Introduction

### 1.1. Background

Anemia affects approximately 17% of older individuals, having a great impact on healthcare systems worldwide [1]. The World Health Organization (WHO) defined anemia in 1968, describing it as hemoglobin levels below 130 gr/dL for men and 120 gr/dL for women [2]. Considering the increasing aging population, particularly in Western countries, the number of anemic individuals is expected to rise significantly [3]. Several underlying disorders associated with aging, including myelodysplastic syndromes, multiple myeloma, malignant blood disorders, and secondary anemia due to gastrointestinal issues, contribute to the increase in chronic anemia frequency in older adults [4].

Anemias can be categorized based on their causes: nutritional deficiencies, bleeding, chronic inflammation, genetic-based anemias (e.g., thalassemia), and anemias because of an abnormal clonal cell population [4]. Anemia has been associated with numerous clinically significant conditions in various epidemiological studies [4]. Low hemoglobin levels are considered a risk factor for cardiovascular diseases, cognitive decline, insomnia, mood disturbances, elevated risk of falls and fractures, and reduced quality of life [5]. Furthermore, chronic anemia in older adults significantly correlates with increased hospitalization frequency and prolonged hospital stays [6]. While current research highlights the importance of hemoglobin levels at diagnosis, further evidence should explore the clinical implications of the declining hemoglobin levels and the progression of anemia [7]. Managing anemia in older adults is challenging, particularly when the cause is unknown or when multiple comorbidities coexist [8]. Age-related changes may impair organ functions, including erythropoietin production, which becomes insufficient to prevent anemia under certain conditions [9]. Targeting anemia in elderly patients requires a multidisciplinary approach and detailed organ functional evaluation [4]. Therapeutic options include supplementation therapy or addressing underlying causes, while in some cases, long-term interventions with erythropoietin or chronic transfusions are mandatory [10]. Achieving optimal hemoglobin concentrations may correlate with better clinical outcomes, as has been demonstrated in several recent studies reporting a significant increase in survival and a decrease in hospitalization risks.

### 1.2. Hematopoietic System, Aging, and Metabolomics Research

Metabolomics is an emerging approach in systems biology that holds a great potential for uncovering insights into the aging processes in many human diseases, including chronic anemia of various etiologies [11]. It offers a highly sensitive and precise approach to evaluating the subtle changes in genetic and protein expression by amplifying them into metabolite forms, using several techniques, including advanced separation methods, state-of-the-art instrumentation, and sophisticated data processing methodologies [12]. Metabolites, the end products of biological processes, offer a quick snapshot of function-related phenotypes, closely associated with physiological conditions [13]. Metabolite formation reflects the interaction between biological and environmental factors, offering a great potential in associating genetic and phenotypic characteristics, gradually transforming into a pivotal tool for assessing an individual’s biological state [14]. During the last few years, research interest has focused on the study of metabolism and metabolomic approaches in correlation with chronic disease management and treatment [15,16].

Hematologic diseases are a field of continuous medical research with several targeted therapies being tested during the last decade. Current research has demonstrated their potential targeting with novel agents and epigenetic therapies, and, furthermore, revealing their potential for being risk factors for other, metabolic or not, chronic diseases of older adults [17,18,19,20]. Interestingly, it has been suggested that significant metabolomic shifts occur during normal and pathological hematopoietic processes, playing a critical role in hematologic disorders’ progression and potentially contributing to drug resistance [21]. Recent studies on animal models indicate that calorie restriction, which directly affects metabolism, may effectively combat age-related diseases; specifically, a recent study which mapped single-cell transcriptomic changes related to fasting’s impact on aging, showing how metabolic interventions may rewire aging processes in various cell types, including blood and bone marrow cells [22,23,24]. Studies have also revealed that aging neutrophils develop a migratory phenotype, infiltrating peripheral tissues and contributing to inflammaging, which calorie restriction may reverse [24]. Furthermore, glucose metabolism influences chromatin structure, transcription processes, and stem cell fate decisions concerning proliferation, differentiation, and dormancy, especially in older adults [25]. RNA sequencing in combination with metabolomics and proteomic approaches has offered valuable insights into the behavior of hematopoietic stem cells and progenitor cells, further offering opportunities in determining stem cell lifespan and fate [25,26].

Aging, a natural phenomenon, has intrigued medical researchers for centuries [27]. Recent studies of the aging metabolome have demonstrated a decline in the mitochondrial tricarboxylic acid (TCA) cycle’s intensity and an increase in glycolytic flux during aging [28]. Disruptions in lipid oxidation pathways and energy production in age-related diseases suggest that these changes may play a crucial role in initiating or accelerating the aging processes [28]. It is notable that metabolite level changes during aging show different trends across organs [29]. Thus, metabolomic studies may provide valuable insights into their metabolic states during aging. Establishing an “aging organ metabolite clock” may offer new understanding and treatment options for aging-related diseases, including chronic anemia of the elderly [29]. Chronic anemia of the elderly has various etiologies, including diseases of hematopoietic dysregulation (myelodysplastic syndromes, multiple myeloma), genetic disorders (e.g., β-thalassemia), and leukemias [9].

During the last decade, many advances regarding the treatment of hematologic diseases have occurred, including novel medication for the management of chronic anemia [30]. A common characteristic of myelodysplastic syndromes and multiple myeloma (both of which can present with anemia) is that they are both associated with significantly increased prevalence with increasing age, and at the same time they are characterized by recently evaluated metabolic changes that can be effectively targeted [31]. Furthermore, despite continuous research, treatment options remain limited, and patients’ prognosis remains poor. On the other hand, β-thalassemia, a genetic disease of chronic anemia due to ineffective erythropoiesis, which was characterized by high mortality at an early age, has now become a chronic disease, with a large percentage of thalassemic population being at an older age [32]. Moreover, β-thalassemia is associated with accelerating cellular aging via several mechanisms, leading to poor quality of life due to multi-organ complications [33].

Based on the current knowledge, we chose to explore evidence regarding (i) metabolism alterations and targeting in myelodysplastic syndromes (MDSs) and multiple myeloma (MM), which are the main causes of chronic anemia in older individuals, and (ii) metabolomics and novel metabolic and mitochondrial targeted agents in β-thalassemia, which is both a disease of accelerating cellular aging, and a disease that has not been studied in older adults until the last few years. Treating these diseases requires a multidisciplinary approach and comprehensive evaluation of organ function. This review describes current concepts regarding the aforementioned causes of chronic anemia in older ages, highlighting etiologies, clinical implications, and innovative management strategies.

## 2. The Role of Metabolism Dysregulation in Myelodysplastic Syndromes

### 2.1. Metabolomic Pathophysiology and Pathogenesis

MDSs are a group of hematopoietic clonal disorders, ranging from asymptomatic cytopenia to severe complications, like severe chronic anemia, thrombocytopenia, and neutropenia, that are characterized by bone marrow dysplasia [34]. In approximately 25% of MDS cases, a malignant progression towards acute myeloid leukemia is observed [34]. Recently conducted research has focused on targeting MDS-related genetic mutations, aiming to initiate new treatments (hypomethylating agents, BCL-2 inhibitors), while allogeneic stem cell transplantation remains the main curative option for high-risk patients, although many of them are ineligible due to age restriction or donor unavailability [35]. Interestingly, metabolic reprogramming has been shown to play a crucial role in hematologic malignancies including MDSs, involving changes in glucose, amino acid, and fatty acid metabolism, with specific patterns being observed, which highlights the need for investigating the targeting of these pathways to slow MDS progression and improve patients’ outcomes [36]. The aging hematopoietic system has been extensively studied, revealing variable changes with age in human and animal models, including decreased production of red blood cells and lymphocytes and a significant increase in myeloid cells [37]. These changes are associated with various alterations in the bone marrow including reduced cellularity, dysregulated chemokine and cytokine production, and alterations in the percentage of non-hematopoietic cells within the bone marrow microenvironment [38].

#### 2.1.1. Alterations and Potential Therapeutic Value of Glucose Metabolism

Glucose metabolism varies between the quiescent and active phases of hematopoietic stem cells (HSCs); in the quiescent state, HSCs primarily rely on anaerobic glycolysis for survival, influenced by a hypoxic microenvironment maintained by elevated levels of hypoxia inducible factor-1 (HIF-1), which interacts directly with HSC metabolic processes [39,40]. HIF-1 activates pyruvate dehydrogenase kinases (PDKs), enhancing anaerobic glycolysis and enhancing stem cell differentiation [41]. Moreover, it has been proven that changes in glucose metabolism in hematopoietic stem cell niches may contribute to MDS pathogenesis. Hematopoietic stem cell niches are specialized tissue environments supporting and regulating HSCs, consisting of vascular endothelial cells, osteoblasts, macrophages, monocytes, and mesenchymal stem cells (MSCs) [42]. These niches produce extracellular matrices and cytokines and enable cell interactions to achieve the HSC microenvironment balance [43]. MSCs play an important role in this process by differentiating into adipogenic, osteogenic, and chondrogenic lineages [44,45]. They mainly rely on glycolysis to maintain energy, even in oxygen-rich conditions, similar to cancer cells, although MSCs also use oxidative phosphorylation during proliferation; during MSC differentiation, increased oxygen consumption and decreased lactate production are observed, shifting the main energy source to oxidative phosphorylation [46,47,48]. An imbalance between oxidative phosphorylation and glycolysis may lead to premature MSC senescence and increased reactive oxygen species (ROS) production, thus disrupting the microenvironment of hematopoietic stem cell niches, and elevating the risk of myelodysplasia transformation. In elderly populations, MSCs favor oxidative phosphorylation, causing ROS accumulation and DNA damage, which is a major reason why MDS predominantly affects these populations.

MDSs are complex diseases originating from HSCs, similarly to acute myeloid leukemia. Recently conducted research has indicated that MDS stem cells share significant similarities with leukemic stem cells regarding surface markers and metabolic traits [49]. MDS stem cells, like leukemic stem cells, primarily rely on oxidative phosphorylation for survival, differing from normal HSCs [50]. Leukemic stem cells depend on amino acid metabolism to maintain oxidative phosphorylation at normal levels and present decreased capacity to efficiently metabolize fatty acids or glucose [51]. During the oxidative phosphorylation process, mitochondrial ROS production is conducted, elevating the probability of developing hematopoietic disorders [52]. Moreover, recent studies investigating the glycolytic activity in MDS blast cells suggest the presence of the Warburg effect; it seems that these cells show a preference for glycolysis in order to generate ATP even in high-oxygen conditions, resulting in elevated glucose consumption and lactate production [53]. The distinct differences in glucose metabolism between normal HSCs and MDS-affected cells offer several opportunities to design novel therapeutic targets for these unique metabolic characteristics.

#### 2.1.2. Alterations in Amino Acid and Fatty Acid Metabolic Changes

Except for glycolysis, metabolomic techniques have also revealed other metabolites’ roles in MDS pathogenesis and progression. To begin with, hematologic malignancies heavily rely on oxidative phosphorylation primarily using glutamine, a phenomenon termed “glutamine addiction” [54]. Glutamine is converted into glutamate, a reaction catalyzed by glutaminase (GLS), which further transforms into a-ketoglutarate (a-KG) in mitochondria fueling the TCA cycle and antioxidant glutathione synthesis [55]. This process enhances ATP production and cell integrity [55]. Studies demonstrate overexpression of GLS1 in MDSs, contributing to MDS transformation into AML, thus acting as a potential marker of poor prognosis [56]. Moreover, tryptophane catabolism into kynurenine via the enzyme indoleamine 2,3-dioxygenase may be a novel target to inhibit MDS progression, as in MDS, dysregulation of tryptophan break down may inhibit hematopoietic stem cell expansion, contributing to blood cytopenia [57,58]. Fatty acid metabolism is also a key pathway in hematologic malignancies pathogenesis, including MDS; MDS cells show altered lipid profiles, with fatty acids playing various roles in MDS progression to AML [59]. Increased levels of myristic and stearic acids may serve as novel markers for monitoring disease progression from pre-leukemic conditions to AML transformation [60]. Genetic alterations in metabolic enzymes, mainly isocitrate dehydrogenase (IDH), are also crucial in the development of MDSs. IDH is essential for converting isocitrate to α-ketoglutarate (α-KG) through oxidative decarboxylation, protecting human cells from oxidative damage via this process [61,62]. IDH mutations are associated with the production of the oncometabolite D-2-hydroxyglutarate (2-HG) [62]. Mutant IDH1 and IDH2 convert α-KG into 2-HG, causing its accumulation, which inhibits oxoglutarate dehydrogenase and reduces succinyl-CoA production, leading to anemia [63]. Elevated 2-HG levels also impede α-KG dependent dioxygenases (histone and DNA demethylases) dysregulating the cell’s epigenetic state [61]. This process results in hypermethylation of histones and DNA, inhibiting cellular differentiation and maturation [64]. Although these mechanisms have not been yet fully elucidated, IDH mutations in higher-risk MDSs are considered to lead to AML malignant progression, being a potential therapeutic target [65,66].

#### 2.1.3. Iron Metabolic Alterations

Iron overload is characterized by excessive tissue siderosis and has been associated with the pathophysiology of many malignant diseases [67,68]. In MDS, iron accumulation is a result of multiple factors, mainly ineffective hematopoiesis, frequent blood transfusions and hepcidin dysregulation [69]. Iron overload is associated with poorer patient outcomes, partly due to its toxicity to the cardiac system [69]. Under normal circumstances, the human body regulates iron levels due to the potential toxicity of iron accumulation [70]. Iron is recycled from aged and dead red blood cells, with macrophages in the reticuloendothelial system reclaiming iron supplies by breaking down hemoglobin [71]. Hepcidin is a peptide produced primarily by hepatocytes and is the key hormone in maintaining iron balance by controlling ferroportin expression, which exports iron from human cells [72]. Thus, decreased intestinal iron absorption is achieved in situations in which ferroportin is downregulated [73].

MDS patients with excess iron are also at a higher risk of developing secondary acute myeloid leukemia due to potential correlations between hemosiderosis and genetic mutations including TET2 and ASXL1, which predispose to MDS progression [74]. Furthermore, increased ROS levels in MDS patients with iron overload may cause DNA damage through oxidative stress [74]. Iron levels upregulate hepcidin via the BMP-SMAD metabolic pathway, while inflammation and aging also increase hepcidin through the IL-6-JAK-STAT pathway, potentially causing inflammation-associated anemia [75]. On the other hand, hypoxic environment and anemia induce erythroferrone (ERFE) production, which reduces hepcidin secretion [76]. Hepcidin levels vary across MDS types, with higher levels being found in higher-risk MDSs and lower levels being found in lower-risk MDSs [69]. Lower-risk MDS patients with SF3B1 mutations present inappropriately low hepcidin levels, resulting in parenchymal iron loading [77]. Moreover, these mutations produce a variant of the ERFE gene that suppresses hepcidin transcription, contributing to decreased hepcidin levels and susceptibility to iron overload [78]. Targeting hepcidin and ERFE is a promising therapeutic strategy for iron overload prevention and improving survival rates [78].

### 2.2. Metabolism-Targeting Approaches in MDS Therapeutics

As previously mentioned, leukemic blasts rely heavily on glycolysis, leading to the development of anti-tumor agents targeting key glycolytic enzymes. Thus, glycolytic enzyme inhibition may serve as a potential therapeutic approach to enhance MDS prognosis [79]. Hexokinase (HK) is crucial for the first step of glycolysis and has three isoforms: HK1, HK2, and HK3 [80]. While HK1 and HK2 are widespread, HK3 is primarily found in myeloid cells and presents significantly increased levels in MDS patients [80]. Although research on HK levels in MDS cells is limited, HK inhibitors [e.g., 3-Bromopyrvate (3-BrPA) and 2-Deoxy-D-glucose (2-DG)] have shown anti-tumor effects [80]. Moreover, the combination of 3-BrPA with rapamycin (RAPA) has been an effective approach in preventing acute graft-versus-host disease by targeting HK and mTOR pathways in allo-HSCT patients, offering a novel treatment option to enhance MDS prognosis [81]. Pyruvate kinase (PK) facilitates the final glycolysis step, producing pyruvate and ATP [80]. Among PK isoforms, PKM2 is predominant in tumor cells and is associated with the proliferative and differentiative capacity of myeloid cells, enhancing the development of hematopoietic malignancies via SUMOylation processes [18,82]. PKM2 overexpression in AML is linked with poor prognosis enhancing leukemic cell survival, especially those carrying nucleophosmin (NPM1) mutations, which, although rare in MDS, are considered vital for their progression to AML [83]. Micheliolide (MCL), derived from michelia plants, effectively suppresses leukemia by initiating irreversible PKM2 tetramerization [84]. Its prodrug, ACT002, is currently being tested in clinical trials for glioma and shows a promising new target for MDS treatment as well [84].

**Figure 1:**
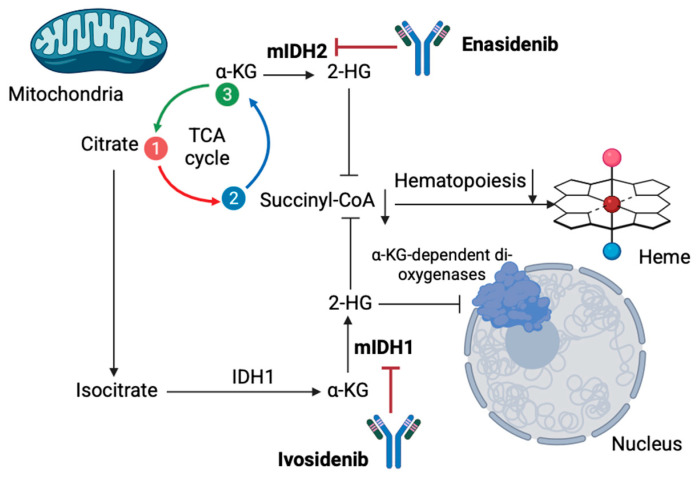
Metabolic targeting in MDS with monoclonal antibodies Enasidenib and Ivosidenib. Ivosidenib and Enasidenib demonstrate potential for MDS treatment by eliminating oncometabolite 2-H by targeting mIDH1 and mIDH2, respectively. As a result, normal hematopoiesis in MDS is restricted [α-KG: α-Ketoglutaric acid; mIDH1/2: mutated isocitrate dehydrogenases 1/2; 2-HG: 2 hydroxyglutarate; TCA: trichloroacetic acid] (the figure was created using BioRender software version 04, License # OE28U5DG3U).

Moreover, other new designed non-glycolytic agents have been recently proposed as potential targets for improving the outcomes of MDS patients. Telaglenastat (CB-839) is a glutaminase inhibitor currently being tested in the NCT03047993 clinical trial for treating MSD, along with azacytidine [85]. Results from the trial’s phase I demonstrated that the combination is promising in terms of safety and efficacy, which was anticipated given the fact that inhibiting glutamine catabolism in MDS cells may induce mitochondrial apoptosis without affecting normal hematopoietic progenitors [85]. Additionally, IDH mutations are considered key components in MDS progression, making IDH1 and IDH2 promising therapeutic targets, especially in high-risk cases [86]. Ivosidenib and Enasidenib, which are well-tested, FDA-approved drugs for relapsed/refractory IDH-mutated AML, show great potential for MDS targeting by eliminating oncometabolite 2-HG [87] (Figure 1). Ivosidenib has been approved for targeting relapsed/refractory IDH1-mutated MDS based on the results obtained from the AG120-C-001 MDS sub-study, which demonstrated complete response rates of approximately 40% and a median overall survival of 35.7 months [88,89]. In treatment-naive high-risk MDSs, using a combination schema of azacytidine and ivosidenib resulted in an overall response rate of 78.3%, with median overall survival, however, not reached after 25.2 months of follow-up [88,89]. The PyramIDH phase III clinical trial is currently comparing single-agent hypomethylating agents and ivosidenib in a frontline setting [88]. Enasidenib is approved for relapsed/refractory IDH2-mutated AML and has shown promising results in MDS as well [90]. A phase I trial reported a 53% overall response rate with favorable responses in 46% of patients previously treated with HMA [90]. A subsequent phase II trial for MDS patients evaluated the safety and efficacy of an azacytidine and enasidenib combination for treatment-naïve patients and enasidenib monotherapy for those pretreated with hypomethylated agents [91]. The combination schema demonstrated a 35% overall response in 52% of the patients, with a median response time of 4.6 months [91]. For MDS patients not responding to IDH inhibitors, Olaparib, a PARP inhibitor, also showed significant potential according to recent published evidence [87].

Iron chelation treatment reduces reactive oxygen species and eliminates their harmful effects in MDS patients [92]. Chelation treatment with Deferasirox has shown efficacy in low-risk MDS, while there is evidence for the efficacy of Deferiprone and Deferoxamine in these cases [93]. Novel targeted agents, including hepcidin agonists, are being evaluated, aiming to decrease iron overload by restoring hepcidin levels and reducing iron absorption in MDS patients. Agents targeting the hepcidin–ferroportin axis, including PR73 and minihepcidins, have shown potential in β-thalassemia and hereditary hemochromatosis, while these agents (specifically rusfertide and vamifeport) are undergoing clinical trials for polycythemia vera and sickle cell disease, respectively [94]. Given the similarities in the pathophysiology of malignant transformation due to reactive oxygen species in all these pathologies and in MDS, further research is needed in order to evaluate the aforementioned drugs’ potential in MDS therapeutic evaluation [95].

Potential therapeutic options targeting metabolism in MDS are summarized in Table 1.

In the context of iron metabolism and regarding serum ferritin levels, the medical literature has reported a wide range of clinical findings suggesting an association between hyperferritinemia and MDS pathogenesis. Many studies suggest that serum ferritin levels could serve as a prognostic indicator; however, inconsistencies in the ferritin thresholds used to define “hyperferritinemia” and the lack of consistently significant results highlight the need for further research in this field [96]. Most studies focus on survival outcomes, generally indicating that hyperferritinemia is associated with worse survival metrics; nonetheless, there is no clear pathogenetic mechanism between serum ferritin levels and the incidence of disease relapse or progression [97,98]. Subgroup analyses in recent studies posed additional challenges, especially given the heterogeneity of MDSs: for example, conflicting results have been reported in event-free survival (EFS) between low- and intermediate-risk patients compared to high-risk groups [96]. These discrepancies underscore the potential interaction between serum ferritin levels and other patients’ factors (comorbidities, risk classification). Variability in model variables and statistical adjudgments further complicate cross-sectional comparisons. To enhance comparability across studies and populations, future research may utilize standardized methodologies that minimize variation, while additional studies are also necessary to explore the potential relationships between SF levels and the pathogenesis of MDSs.

## 3. Multiple Myeloma

### 3.1. The Need for Novel MM Therapeutic Targets in the Elderly

Multiple myeloma (MM) accounts for approximately 10% of hematologic malignancies and is characterized by terminally differentiated effector cells which produce monoclonal immunoglobulins as a response to immune stimuli [99]. MM cells originate from antibody-secreting B lymphocytes [100]. Clonal evolution occurs as a result of genetic alterations in these cells, including translocations and hyper-diploidy, followed by secondary genetic events, finally resulting in malignant cell formation [101]. MM pathophysiology is complex, involving alterations in the tumor microenvironment, deregulated signaling pathways, and genomic instability, resulting in the use of multiple therapeutic strategies over the past decade: immunomodulatory drugs, chimeric antigen receptor T-cell therapy, autologous hematopoietic stem cell transplantation, antibody–drug conjugates, and proteasome inhibitors [102,103]. MM primarily affects older adults [104]. Treatment outcomes for elderly patients are often hindered by comorbidities and increased vulnerability to therapy-related events [104]. Recent advances in immunotherapies (e.g., CD38 monoclonal antibodies), along with emerging immune-oncology agents, are associated with promising results in treatment [104].

Despite the variety of advancements in MM therapeutic evaluation, MM remains an incurable malignancy, with a substantial number of cases experiencing relapse and developing resistance to current treatment options [105]. Proteasome inhibitors (PIs), which have been explored in terms of their involvement in the ubiquitin–proteasome system, have demonstrated significant therapeutic effects on MM, acting by inhibiting the degradation of misfolded proteins and leading to their accumulation in the endoplasmic reticulum (ER) and overwhelming ER stress [106]. PIs also disrupt key metabolic pathways (reducing glucose uptake, decreasing mitochondrial energy production, enhancing lipid catabolism, and lowering intracellular amino acids (glutamine) [107]. Based on the clinical experience of affecting MM metabolism using PIs, the most effective frontline treatment in MM, research has recently focused on targeting metabolic pathways as a potential treatment option for MM therapeutic evaluation [107]. Moreover, a critical clinical question is whether the new therapies (immunotherapy, immune-oncology agents) can be applied to elderly patients; thus, novel strategies should be explored in this patient population [104].

### 3.2. Targeting Glucose Metabolism in MM

Glucose is important for cell viability; it provides energy in the form of ATP and, depending on oxygen concentration, its metabolism results in either carbon dioxide or lactate [108,109]. Nearly a century ago, cancer cells favoring glycolysis over oxidative phosphorylation regardless of oxygen presence were discovered, a situation termed the “Warburg effect” [109]. The Warburg effect enhances rapid ATP synthesis, fuels biosynthetic pathways, alters tumor microenvironments for immune evasion, and connects glycolysis and oxidative phosphorylation through pathways involving HIF-1, AMPK, and oncoproteins (e.g., RAS, MYC) [110]. Targeting glucose metabolism may show a significant potential in MM therapeutic evaluation [111]. As with all malignancies, MM heavily relies on glucose, meaning that radiolabeled glucose analogs (e.g., 18F-fluorodeoxyglucose) have several roles in diagnostic imaging for MM, while MM cells have high demands for glucose, which is essential for their growth and chemoresistance [111].

Regarding MM therapeutics, inhibiting hexokinase (HK) activity, which converts glucose to glucose-6-phosphate, driving tumor glycolysis, by using antisense oligonucleotides, may effectively inhibit HK1-HK2+ MM cell proliferation, while combining HK2 inhibition with partial oxidative phosphorylation inhibitions may show promising results in eliminating MM progression [112]. Targeting pyruvate dehydrogenase kinase (PDK), which plays a pivotal role in the Warburg effect regulating glycolysis and oxidative phosphorylation, may restore acetylo-CoA supply to the Krebs cycle, leading to apoptosis by increasing mitochondrial oxidative stress [113]. Dichloroacetate, a PDK inhibitor, reactivates pyruvate dehydrogenase activity, restoring mitochondrial function and serving as a potential target [114]. Inducing reactive oxygen species (ROS) may promote the apoptosis of MM cells, alter metabolic phenotype by inhibiting the Warburg effect, and enhance sensitivity to monoclinic antibodies (e.g., bortezomib) [115]. Targeting the Warburg effect may involve a variety of molecular agents; for example, PRL-3, a phosphate agent that may enhance oxidative phosphorylation and ATP production, and FOXM1, a suppressor of MM cell growth, offering potential therapeutic strategies [116].

Human glucose transporters (GLUTs) facilitate hexose transport. Unlike solid tumors, where GLUT1 is prominent, MM cells predominantly express constitutive GLUT4 at the cell surface, supporting basal glucose consumption and Mcl-1 expression [117]. The FDA-approved HIV protease inhibitor Ritonavir has been shown to inhibit GLUT4-dependent glucose uptake, reducing MM proliferation, and increasing chemosensitivity [118]. This finding has spurred the development of isoform-specific glucose transporter inhibitors [118]. By combining ritonavir with metformin, a mitochondrial complex I inhibitor, MM cell sensitivity is enhanced by shifting metabolism towards glutamine reliance, showing efficacy in vitro and in vivo and serving as a promising therapeutic option to target metabolic plasticity and prevent chemoresistance [118]. Moreover, multiple molecular targets involved in MM aerobic glycolysis have been identified. For example, phosphatase of regenerating Liver-3 (PRL-3), overexpressed in MM, promotes the Warburg effect independently of its phosphatase activity and increases glycolytic and amino acid synthesis enzymes; downregulating glycine decarboxylase may counteract PRL-3 mediated metabolic reprogramming [119]. Forkhead Box Protein M1 (FOXM1), a transcription factor regulating cell cycle genes, also promotes MM glycolysis and oxidative phosphorylation [120]. Targeting FOXM1 with specific inhibitors such as NB73 may inhibit its degradation and decrease MM cell growth and differentiation, being another promising therapeutic option [120].

### 3.3. Targeting Lipid Metabolism in MM

Targeting lipid metabolism has been recently studied in a great variety of (malignant or not) diseases in populations of all ages [121,122]. Regarding malignant diseases, highly proliferative cancer cells require substantial amounts of exogenous lipids and lipoproteins to maintain their growth [123]. They activate their own lipid synthesis pathways, including lipogenesis and cholesterol biosynthesis, in order to fulfil this demand [124]. Lipogenesis is driven by the elevated activity of fatty acid synthase (FAS), which is observed in a variety of tumors [125]. De novo fatty acid synthesis, along with lipid uptake and suppression of fatty acid oxidation, contributes to lipid storage, which is considered a key adaptive mechanism of tumor survival [126]. Inhibiting FAS activity by blocking its formation is considered a promising therapeutic strategy in several malignancies [127]. Cancer cells also store excess lipids and cholesterol in specific structures termed lipid droplets (LDs), the presence of which is considered a marker of poor prognosis and chemoresistance [128]. Similar alterations in lipid metabolism have been demonstrated in MM, where they may promote tumor cell growth and survival by influencing the tumor microenvironment via abnormal bone marrow adipocytes [129]. Hyperlipidemia, and particularly elevated levels of immunoglobulin A (IgA), have been reported in MM patients [130]. MM cells depend on exogenous cholesterol for survival, with low-density lipoprotein (LDL) cholesterol acting as an antiapoptotic factor [131].

Statins are 3-hydroxy-3-methylglutaryl-CoA (HMG-CoA) reductase inhibitors being the main lipid-lowering agents [132]. Their use in MM patients has been reported to reduce overall mortality by promoting apoptosis and cell growth [132]. Statins inhibit the conversion of HMG-CoA into mevalonic acid, resulting in decreased intracellular cholesterol levels, and, furthermore, they promote the cleavage of sterol regulatory element binding proteins (SREBPs) from the endoplasmic reticulum; SREBPs translocate to the nucleus and upregulate genes encoding LDL receptors [133]. Moreover, in MM patients, lipids from specialized membrane domains known as lipid rafts, may facilitate the recruitment of signaling proteins and promote signal transduction, and are currently being investigated as therapeutic targets in MM, using the phospholipid ether edelfosine [134]. Edelfosine directly targets and accumulates within MM cell membrane rafts, inducing apoptosis by co-clustering rafts with death receptors [134]. Disrupting cholesterol in the membrane dysregulates edelfosine’s uptake and inhibits its apoptotic effects [134]. Reintroducing cholesterol restores drug-induced apoptosis, underscoring cholesterol’s role in raft integrity and drug efficacy [135]. Another potential target in MM is the enzyme ELOVL6, which is responsible for elongating very-long-chain fatty acids and is transcriptionally regulated by SREBP1 [136]. In MM, SREBP1 expression is positively correlated with ELOVL6 levels, and alterations in ELOVL6-related lipids are associated with response to bortezomib and drug resistance [136]. Notably, ELOVL6 expression is reduced in MM patients and cell lines resistant to bortezomib, and thus depleting ELOVL6 expression re-sensitized cells by increasing factors (ceramide species) dependent on ELOVL6, indicating its role in mediating bortezomib resistance [136].

MM, like other malignancies, heavily relies on interactions with the tumor microenvironment (TME) for survival, progression, and chemoresistance [137,138]. Bone marrow adipocytes, which comprise approximately 70% of the bone marrow volume, expand with aging [137]. The dysregulated crosstalk between bone marrow adipocytes and MM cells enhances tumor growth, survival, chemoresistance, and MM bone disease [139]. Adipocytes regulate cellular metabolic plasticity; mainly, adiponectin plays a critical role in glucose metabolism and insulin resistance, having, at the same time, tumor-suppressing effects [140]. Thus, decreased expression of adiponectin favors tumor progression and osteolysis. MM cells may induce lipolysis in bone marrow adipocytes, uptaking free fatty acids (FFAs); at decreased FFA concentration, they may enhance cell proliferation, whilst at increased concentration, lipotoxicity occurs [141]. Additionally, MM cells may alter gene expression and cytokine secretion of adipocytes, inducing a senescent-like phenotype that supports resistance to dexamethasone-induced cell cycle apoptosis [142]. Based on these interactions, potential therapeutic choices arise; the apolipoprotein peptide mimetic L-4F enhances adiponectin levels, reducing tumor burden, prolonging survival in MM and preventing MM bone disease [143].

### 3.4. Targeting Amino Acid Metabolism in MM

Dysregulated amino acid metabolism is associated with carcinogenesis, and is decreases amino acid intake, inhibiting tumor growth, serving as a promising therapeutic target [144]. Proteasome inhibitors deplete intracellular amino acids, causing mitochondrial damage and activating the general control non-depressible 2 (GCN2) pathway (an important sensor of amino acid starvation), which may lead to chemoresistance [107]. GCN2 activation is also associated with MYC signaling and mTORC1 inhibition via leucine deprivation [145]. In MM patients, genes related to amino acid metabolism are overexpressed and hypermethylation of asparaginase synthase occurs at a significant level, representing a potential therapeutic target [146]. Targeting amino acids with Erwinia chrysanthemi-derived asparaginase synthase has demonstrated promising effects enhancing mitochondrial ROS-mediated cell death [146]. Another potential target is glutamine metabolism; glutamine metabolism alterations including increased use of gut bacteria (e.g., Klebsiella pneumoniae) lead to MM progression and dysregulate the bone marrow environment [146]. Targeting glutamine with compounds like L-asparaginases or by monitoring metabolites (e.g., 2-HG) may offer potential strategies for therapeutic evaluation [146].

Non-essential amino acids, including serine and glycine, are vital for proliferation of MM cells, especially because they activate de novo synthetic pathways which contribute to chemoresistance, for example, the serine synthesis pathway [107]. Serine also influences megakaryocyte function and thrombocytopenia, a common complication in MM [107]. MM cells are characterized by matrix metalloproteinase 13 (MMP13) secretion, which upregulates glycine levels in the bone marrow and supports tumor growth through pathways including glutathione and purine synthesis [107]. Targeting amino acid transporters and synthesis pathways is a potential therapeutic option for MM treatment [107].

**Figure 2:**
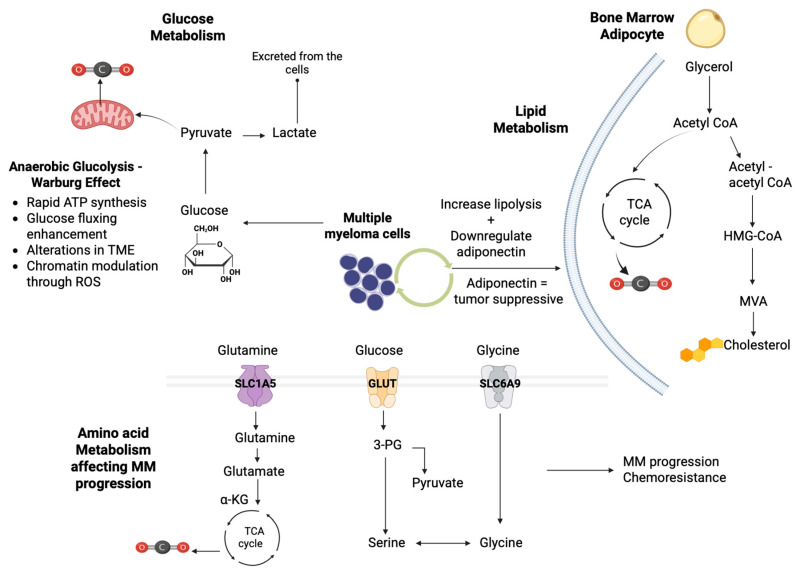
Illustration of the key metabolic pathways involved in MM progression. Glucose metabolism: MM exhibits the Warburg effect, demonstrating increased glucose uptake and rapid ATP synthesis through glycolysis, leading to lactate production from pyruvate and involving significant chromatic modulation through ROS signaling. Lipid metabolism: In the bone marrow adipocytes, lipid metabolism is crucial for MM progression by increasing lipolysis downregulation of adiponectin. Acetyl CoA feeds into the TCA cycle, contributing to cellular energy production and enhancing MM cell proliferation. Amino acid metabolism: Glutamine is transported via SLC1A5 into the cells, while glucose-derived serine and glycine are transported through GLUT and SLC6A9 transporters, respectively, contributing to MM progression and chemoresistance [α-KG: α-ketoglutarate; ATP: adenosine triphosphate; HMG-CoA: 3-hydroxy-3-methylglutaryl-coenzyme A reductase; MVA: mevalonate; SLC1A5/6A9: Solute Carrier Proteins 1A5/6A9; TCA: tricarboxylic acid cycle; TME: tumor microenvironment] (the figure was created using BioRender software version 04, License #CL28U674DW).

An illustration of how glucose, lipid, and amino acid metabolism affect MM progression is provided in Figure 2.

### 3.5. The Role of Iron Metabolism in MM

Considering that, in hyper-inflamed neoplasms, including MM, the tumor microenvironment is iron-enriched due to release from proinflammatory cells (e.g., tumor-infiltrating macrophages and granulocytes), research has been conducted regarding the role of hyperferritinemia in MM prognosis [147]. Hyperferritinemia may promote neoplastic progression by inducing immune suppression, including B and T lymphocyte apoptosis, and impairing antigen-presenting cell (APC) activity [148]. Current research indicates that ferritin may be a negative prognostic factor in MM, with elevated levels correlating with severe disease and elevated expression of markers of systemic inflammation (CRP), IL-6, and LDH [149]. Furthermore, elevated ferritin levels inhibit tumor necrosis factor-induced apoptosis, inducing MM progression [150]. An Austrian study suggested that high pre-transplantation serum ferritin levels are an independent negative prognostic factor for progression-free survival (PFS) and overall survival (OS) in MM patients eligible for autologous stem cell transplantation, while a more recent study confirmed these results in transplantation-ineligible patients revealing a specific enrichment f S-phase genes in MM patients with hyperferritinemia [147,150].

The role of ferritin in promoting MM cell proliferation has recently been examined: patients with hyperferritinemia exhibited significantly increased bone marrow plasma cells compared to controls, while a reduced number of NK CD38 cells in the same group was observed, which is considered a notable finding, given these cells’ roles in tumor surveillance and antibody-dependent toxicity [151]. Moreover, an integrated bioinformatics analysis of three available single-cell RNA sequencing datasets involving plasma and immune cells from MM patients revealed a significant association between high-ferritin-correlated gene expression and alterations in the immune microenvironment, specifically decreased NK cells and increased monocytes [147]. This evidence combined highlights the importance of evaluating ferritin in the assessment and monitoring of MM patients and propose ferritin as a potential novel target for MM therapy, paving the way for future combination studies using iron chelation to enhance immunomodulatory treatments or the use of monoclonal antibodies [152]. Further research is mandatory to validate ferritin as a prognostic biomarker and to clarify its potential as a therapeutic target for improving MM patient outcomes. 

## 4. B-Thalassemia

### 4.1. B-Thalassemia Is a Disease Model of Accelerating Cellular Aging

Despite the advances in iron chelation therapy, iron overload remains the main cause of mortality in β-thalassemia major, primarily due to iron accumulation in vital organs, which results in tissue damage and organ failure, predisposing to premature aging of organ systems of individuals with thalassemia [153,154,155]. In physiological conditions, iron transport is tightly associated with iron-responsive proteins, with transferrin binding circulating non-heme iron for cellular uptake via transferrin receptors [156]. However, in iron overload states, excess non-transferrin-bound iron (NTBI) circulates freely, presenting high toxicity, as it readily enters the cell, increasing the levels of labile cell iron that catalyze the formation of ROS, causing extensive cellular damage [157]. Iron overload also results from macrophage recycling, excessive intake, genetic factors, and hemolysis from systemic transfusions [158]. The accumulation of NTBI correlates with increased oxidative byproducts and reduced antioxidant capacity [158]. Iron’s redox properties enhance the production of ROS via the Fenton reaction, contributing to oxidative stress that cause damage to protein, lipids, and DNA [158]. The presence of unpaired α-globin genes in severe β-thalassemia accelerates ROS production, further exacerbating cell injury [159]. This oxidative environment enhances premature cellular aging, notably in immune cells (e.g., T lymphocytes) which exhibit shortened telomeres and reduced proliferation, especially marked by a decrease in CD28 [160]. Senescent T cells impair immune responses and contribute to the higher infection risk in individuals with β-thalassemia [160].

It is easily understood that chronic oxidative stress in β-thalassemia patients effectively models immunosenescence, similar to aging and chronic viral infections, which drive immune aging [161]. Targeted therapies modulating senescent immune cells and boost antioxidant defenses may enhance immune resilience and improve patients’ outcomes [162]. Characterizing senescent lymphocytes’ phenotypes, including telomere length, p53 levels, ROS activity, and surface markers (including CD57 and CD100), is needed in order to gain a better understanding of immune aging in thalassemia, aiming to investigate novel therapeutic approaches to enhance immune function, reduce infection-related morbidity, and improve quality of life in individuals with β-thalassemia [163]. Moreover, since β-thalassemic patients are characterized by premature organ aging and damage, mainly via iron accumulation, they are an important population in terms of investigating metabolic disturbances, providing evidence which may be used in elderly or immunosenescent populations.

### 4.2. Metabolic Alterations in β-Thalassemia Patients

Current evidence suggests that in β-thalassemia, several metabolites are notably altered, reflecting underlying metabolic disturbances. Characteristically, a recent study comparing serum metabolites between β-thalassemia patients and healthy controls regarding their metabolomic profile, using a model based on 40 significant metabolites, revealed significant differences, indicating a metabolic shift from normal states [164]. Among upregulated metabolites, the geraniol-a monoterpenoid with anti-tumor and antioxidant capacity was found to be increased, probably as a response to oxidative stress and inflammation, which are especially increased in elderly individuals [165]. Palmitic acid, a common saturated fatty acid which is involved in energy production and apoptosis, demonstrated elevated levels, potentially contributing to early red blood cell degradation [164,166]. Lactic acid serum levels were also increased, which may be attributed to increased anaerobic glycolysis due to organ hypoxia in anemic patients, which increases with age, or impaired liver function, associated with chronic iron accumulation [164]. Sucrose, a disaccharide broken down into glucose and fructose, is elevated, indicating metabolic dysregulation, and potentially associated with the high prevalence of diabetes in β-thalassemia [164,167]. Additional metabolites including citronellal formate, triethanolamine, and phosphoric acid are also upregulated [164].

On the other hand, several metabolites were proven to be decreased in β-thalassemia patients. Hexadecane, which is an anti-inflammatory and antioxidant metabolite, demonstrates reduced levels, possibly due to increased utilization [164]. Glycerol, which is considered essential in lipid metabolism and energy production, is also decreased, due to increased consumption under conditions of metabolic stress [168]. Stearic acid, another important (for membrane integrity and cell signaling) fatty acid, is decreased, further weakening red blood cell membranes, which are also weakened in older individuals with thalassemia [164]. Ethylene glycol, a compound involved in biological functions is decreased potentially exacerbating oxidative stress and infection susceptibility [164]. Metabolic pathway analysis has revealed significant dysregulation in metabolic pathways including fatty acid elongation, glycolysis, gluconeogenesis, pyruvate metabolism, glycerophospholipid, galactose, and sucrose metabolism [164]. It is worth noting that fatty acid biosynthesis and galactose metabolism are altered in both directions: fatty acid biosynthesis demonstrates elevated palmitic acid and decreased stearic acid, suggesting a compensatory regulation [169]. Regarding the aforementioned dysregulation in galactose metabolism, it is worth noting that increased sucrose affects galactose metabolism, but decreased glycerol indicates incomplete compensation. This imbalance, especially in galactose metabolism, may be associated with diabetes, a common complication in β-thalassemia [164,170]. Overall, the main metabolic pathways affected in individuals with β-thalassemia include glycerolipid, pyruvate, galactose, sucrose, and fatty acid metabolism, with the basic metabolites responsible for this deviation being glycerol, lactic acid, sucrose, and palmitic acid.

### 4.3. Metabolite Patterns Implicated in the Different Therapeutic Options for β-Thalassemia

#### 4.3.1. Chronic Transfusions

Chronic transfusions, which become more frequent in elderly patients, are associated with metabolic shifts towards healthier profiles [171]. However, studies show conflicting results regarding glutathione metabolism and antioxidant levels post-transfusion, with some studies reporting a decrease in glutathione and antioxidant levels, possibly due to oxidative stress during storage and transfusion, and some studies reporting an increase, possibly due to the introduction of fresh red blood cells contributing to the creation of new antioxidants or correcting antioxidant deficiencies [172]. This discrepancy may arise from variations in study design, or differences in patient cohorts, transfusion methods, and frequency and timepoint of post-transfusion measurements; thus, further research is needed to clarify these outcomes. Red blood cells post-transfusion show higher levels of metabolites such as 6-phosphogluconate, purine monophosphates, and catecholamines [173]. Systemic hypoxia markers decrease and glycolytic activity increases, as demonstrated by elevated glycolytic metabolites, with additional changes including increased free fatty acids and ribose phosphate, and decreased levels of acylcarnitine and pyridoxamine [174].

#### 4.3.2. Hydroxyurea

Hydroxyurea is an antimetabolite and a monohydroxylated urea (hydroxycarbamate), which, similarly to other anticancer medication, may induce fetal hemoglobin production [175]. It is mostly used to treat patients with non-transfusion-dependent β-thalassemia [175]. Regarding hydroxyurea metabolic patterns, a recent study, comparing the serum metabolite patterns of β-thalassemia patients before and after 6–12 months of hydroxyurea treatment, demonstrated that most metabolites presented similar regulation patterns post treatment, though their intensities varied [176]. Firstly, linolenic acid, which is essential for prostaglandin synthesis and cell membrane integrity, was elevated in untreated patients, possibly due to inflammation, oxidative stress and increased red blood cell turnover, while post treatment its levels were normalized, indicating a reduction due to the aforementioned pathological processes [177,178,179]. Palmitic and stearic acids, involved in membrane stability and fatty acid metabolism, were also normalized post treatment, possibly due to improved red blood cell membrane integrity [176]. Glycerol levels, which are indicative of metabolic stress, decreased post treatment, reflecting reduced energy demand [176]. Geraniol, an antioxidant and anti-inflammatory exogenous metabolite, was higher before treatment, correlating with elevated oxidative stress, while its levels declined post treatment [176]. Similarly, M-pyrol levels, associated with urinary tract infections, decreased as hydroxyurea treatment reduced infection risk factors [176]. Other metabolites, including decane, lauryl iodide, and heptadecane, demonstrated similar patterns to healthy controls post treatment, indicating recovery [176].

Metabolism pathway analysis revealed that linoleic acid, glycerolipid, and fatty acid metabolism were mostly affected in patients with alterations in metabolites including linoleic acid, glycerol, and palmitic acid [176]. These changes also affected metabolic pathways including galactose metabolism, fatty acid biosynthesis, and mitochondrial elongation [176]. Interestingly, pathways typically altered in anemia (mainly glycolysis and the pentose phosphate pathway) were not directly implicated in β-thalassemia based on the results of this study, suggesting that the impacted pathways are more specific to disease complications, which significantly increase with age [176]. The linoleic acid pathway, which was reported to be highly responsive to hydroxyurea treatment, is considered of great importance for cardiovascular health and the regulation of inflammatory processes [180]. Increased levels of linoleic acid in untreated patients may be a response to iron overload and increased erythropoiesis, processes which increase with age and are significant factors of mortality in elderly thalassemia patients and contribute to inflammation and blood clotting [181]. Hydroxyurea treatment was shown to normalize this pathway, reducing pathological inflammation and RBC degradation [181]. Similarly, glycerolipids and fatty acid pathways which were upregulated to compensate for increased fatty acid needs in β-thalassemia patients were normalized post treatment, indicating a reduction in metabolic stress [182]. The aforementioned findings, comparing serum metabolites of healthy individuals and pre- and post-treatment β-thalassemia patients, provide valuable insights for disease prognosis, treatment monitoring, and supporting hydroxyurea as an effective therapeutic option, especially in elderly individuals whose metabolomic profile is significantly altered.

#### 4.3.3. Mitapivat

B-thalassemia patients often demonstrate low glutathione levels, decreased hepcidin and antioxidant capacity, and elevated malondialdehyde, indicative of oxidative damage [183]. Oxidative stress further impairs glycolytic pathways. Studies using magnetic resonance spectroscopy have reported that glucose metabolism in red blood cells of β-thalassemia patients is significantly elevated compared to healthy individuals or heterozygotes, even after excluding reticulocyte contributions [184,185]. On the other hand, ATP levels in RBCs from β-thalassemia major are significantly reduced, suggesting a shift in glucose metabolism away from glycolysis and towards the pentose phosphate pathway, likely as a response to oxidative stress [184]. Additionally, the activity and stability of pyruvate kinase, which is a key enzyme of glycolysis, are significantly decreased in transfusion-dependent β-thalassemia, while recent studies on Hbb^th3/+^ mouse models (constructed by deleting both the b^minor^ and b^major^ genes in heterozygosity) have demonstrated that elevated expression of PKM2 and Protein Kinase R (PKR) may act as compensatory mechanisms [186]. These findings suggest that activating pyruvate kinase may reduce oxidative damage by decreasing hemolysis, thus improving erythropoiesis, extending RBC lifespan, and alleviating anemia [186]. Improved erythropoiesis may also normalize iron metabolism and reduce iron overload, offering a potential therapeutic strategy for thalassemia patients [187]. Towards this direction, mitapivat, an activator of pyruvate kinase acknowledged for pyruvate kinase deficiency, demonstrated promising effects in β-thalassemia, decreasing 2,3-DPG levels, further improving hemoglobin oxygen affinity, and reducing hemolysis [187,188]. Metabolomic analysis after 8 weeks revealed that mitapivat affected 85 metabolites, with 15 of them shifting towards normal. It enhances glycolysis, increasing upstream intermediates and end products like pyruvate and lactate, and decreases free fatty acid levels over time [189]. Moreover, it has been associated with increased levels of amino acids, bile acids, glutathione, and pyrimidine metabolites, alongside decreases in glycerate, purine derivatives, and bilirubin [187].

### 4.4. Targeting Mitochondrial Metabolism in β-Thalassemia: A Novel Promising Metabolic-Related Approach

Dysregulation of mitochondrial dysfunction and cell senescence are considered key features of aging and are interconnected [190]. Mitochondrial dysfunction, characterized by reduced respiratory capacity and diminished membrane potential and often accompanied by increased production of ROS, serves as both a cause and a result of cellular aging [190]. These processes are embedded in feedback loops that maintain and reinforce the senescent state [190]. Additionally, the potential of targeting mitochondrial dysfunction associated with senescence is a strategy for antiaging and anti-senescence therapies, especially in populations like β-thalassemic patients, who are characterized by accelerated cellular aging [190].

The association between mitochondrial metabolism and β-thalassemia has not yet been fully elucidated. Excess globin chains’ accumulation in developing red blood cells may directly interact with mitochondria, impairing their functionality [191]. Moreover, ineffective erythropoiesis leads to an erythropoietin-induced expansion of early erythroid precursors, thus elevating the metabolic load on mitochondria, which further results in mitochondrial dysregulation, as these cells attempt to meet the energy needs associated with ineffective erythropoiesis [191,192]. Mitochondrial dysregulation leads to the production of excessive ROS which causes oxidative damage and may further damage mitochondrial elements (membranes, proteins, nucleic acids), impairing cell metabolic functions [193]. Recent studies have shown that isolated CD34+ cells from β-thalassemia patients demonstrate a significantly altered redox state compared to healthy controls, with mitochondrial dysregulation occurring by day seven of differentiation and marked deficits in activity occurring near day ten [193]. At this stage, the elevated mitochondrial count observed in thalassemia cells suggests amplified damage, which aligns with the onset of apoptosis and ineffective erythropoiesis [193]. Moreover, increased autophagic activity has been observed in β-thalassemia erythroblasts during erythropoiesis, alleviating the buildup of excess unbound α-globin precipitated by degrading these toxic proteins, acting as protection from mitophagy (selective degradation of active mitochondria) [194]. This evidence highlights the critical role of erythroid maturation in β-thalassemia and suggests that restoring proper terminal erythroid development might be a promising therapeutic strategy [194].

Mitochondrial-targeted therapies for β-thalassemia have not yet been evaluated in clinical trials; however, ongoing research continues to explore aspects of mitochondrial dysfunction, aiming to improve treatment outcomes in the future. For instance, the recently approved medication luspatercept, which acts as a ligand trap for Transforming Growth Factor (TGF) β-like molecules, enhances late erythroblast differentiation, reduces hemichromes, and alleviates anemia in a dose-dependent manner [195]. The exact metabolic pathways involved in the disease pathogenesis have not been fully elucidated [195]. Conversely, as previously mentioned, mitapivat, a pyruvate kinase activator, has shown promising metabolic effects in terms of improving erythropoiesis and increasing ATP levels, reducing ROS, and supporting mitochondrial clearance [196]. Mitochondria-targeted antioxidants (e.g., MitoQ) aim to specifically neutralize mitochondrial ROS, thereby reducing oxidative stress and improving mitochondrial function [197,198]. In recent in vitro studies, MitoQ has lowered mitochondrial ROS levels and restored the proper function of hematopoietic stem cells, achieving a balance between glycolysis and oxidative phosphorylation [198]. Recent studies have also demonstrated that PGC-1α is a potential target for elevating fetal hemoglobin production [197]. Pharmacological activation of PGC-1α using small molecules including SR-18292 and ZLN005 has been shown to increase HbF levels in human erythroid cells and reduce disease severity in transgenic mouse models [197]. Overall, evaluating the regulation of mitochondrial function and erythropoiesis is crucial, and targeting mitochondrial pathways presents a promising avenue for developing novel treatments for β-thalassemia, especially in elderly populations, in whom organ damage is more severe and response to therapeutic options is poorer.

### 4.5. Targeting Iron Metabolism

Considering that restoring hepcidin may reduce iron absorption and improve erythropoiesis, hepcidin’s role in managing iron levels in thalassemia has been recently studied, especially in conditions in which hepcidin is deeply suppressed [199]. Hepcidin mimetics (e.g., LJPC-401 and PTG-300) have shown promising results, while hepcidin agonist-targeting regulators (TPRSS6) are currently being tested in early-phase clinical trials [200,201]. Ferroportin inhibition is also a promising approach, with novel medication (e.g., VIT-2763) demonstrating potential in reducing serum iron [202]. Erythropoietin (ERFE) inhibition and transferrin-2 (TFR2) inactivation are also explored regarding their effects on anemia and iron overload [203]. However, many challenges regarding the use of hepcidin mimetics as a standard routine practice remain due to difficulties in achieving a stable compound, the need for frequent subcutaneous injections, and the frequency of site reactions [204]. Moreover, it is considered difficult to tune their effect, avoiding iron over-restricted erythropoiesis. Given the fact that hepcidin and its modulators are not limited to iron metabolism and erythropoiesis, it is important to develop long-term studies examining potential positive and negative consequences [204]. It is difficult to define the precise position of hepcidin targeting in the rapidly expanding pipeline of treatment options for thalassemia. Excluding a possibility for a curative effect, we may forecast a relevant role in decreasing disease burden and improving disease phenotype, as has been observed with luspatercept, as monotherapy or combination schema.

## 5. Discussion

The expanding field of targeted therapy in many types of malignant and non-malignant diseases in patients of all ages, has offered new insights into the pathophysiological deregulation caused by these diseases [205,206,207]. Furthermore, many types of novel therapies targeting proteinic molecules, may be implicated in metabolic processes, highlighting the potential of further exploring of metabolic targeting [208,209]. With growing evidence highlighting the complex network of metabolic processes, including lipids, amino acids, and nucleotide metabolism, metabolic pathways serve as key example of how metabolic intermediates and enzymes could offer novel therapeutic targets. Metabolic dysregulation, involving abnormal glucose, amino acid, and fatty acid metabolism is considered crucial in diseases of chronic anemia in the elderly, and serves as a potential therapeutic target in several diseases, with multiple myeloma, myelodysplastic syndromes, and β-thalassemia being representative in terms of metabolic reprogramming and its targeting [107]. While these abnormalities are common in the aforementioned diseases, each one has unique metabolic traits. Herein, we provided an analytical description of recent advances in understanding metabolic abnormalities in myelodysplastic syndromes, multiple myeloma, and β-thalassemia, highlighting significant discoveries related to metabolic dysregulation, and offering insights into the complex metabolic disruptions associated with the diseases.

MDSs are marked by ineffective hematopoiesis that leads to a variety of clinical manifestations, the severity of which increases in older ages. While advances in understanding MDS pathology have led to the development of multiple treatment options, allogeneic stem cell transplantation remains the only potentially curative option for high-risk patients [210]. Metabolic alterations have emerged as significant contributors to the pathogenesis and progression of MDSs, prompting the creation of novel metabolic-targeted treatments, which may be valuable especially in elderly population characterized by metabolic dysregulation, which may worsen disease prognosis [210]. MM is a heterogenous disease with diverse treatments that may extend patient survival. Despite recent advances with new agents, metabolic reprogramming through hypoxia and increased lactate production enhances malignant growth and chemoresistance [211]. MM cells primarily rely on glycolysis and lactate production; thus, targeting glucose metabolism presents potential therapeutic opportunities [107]. Moreover, combining antimyeloma therapies with medication targeting lipid and amino acid metabolism may be a promising future strategy [107]. In β-thalassemia patients, who lose their response to therapy as they get older, and are characterized by premature cell senescence, which leads to metabolic dysregulation, targeting metabolism has already been tested in clinical trials, initiating novel agents, namely luspatercept and mitapivat, with great results in terms of safety and efficacy [187,188,195].

Combining therapies to target multiple pathways simultaneously may overcome the redundancy and compensatory mechanisms in the metabolic pathways in diseases of chronic anemia. However, combination metabolic-targeting therapies may bring their own challenges, including the increased risk of toxicity and side effects [212]. Optimizing dosing and scheduling requires extensive testing, yet specifically myelodysplastic and MM cells may still develop resistance through genetic mutations and epigenetic changes [213]. In order to improve the effectiveness of metabolism-targeting combination therapies, future strategies may involve designing combinations through high-throughput screening and computational modeling, which may enhance therapeutic outcomes with minimal side effects [214]. Personalizing therapies based on individual genetic and metabolic profiles can optimize outcomes and minimize adverse events. Identifying reliable biomarkers for metabolic targeting is definitely challenging, while metabolic biomarkers may also change in response to therapy and disease progression, necessitating robust real-time monitoring methods. Some biomarkers are difficult to measure non-invasively, which limits their clinical utility. To our knowledge, there are no established common metabolomic issues that affect anemia itself. However, a potential signaling pathway that could be targeted in cases of chronic anemia of various etiologies is the cd73-mediated conversion of AMP to adenosine, which is enhanced by erythrocyte-specific production of sphingisine-1-phosphate (S1P) [215]. These mechanisms promote the production of 2,3-biphosphosglycerate, a key allosteric modulator that reduces hemoglobin’s affinity to oxygen, enhancing chronic inflammation and tissue damage [216]. Recent studies have identified erythrocyte equilibrative nucleoside transporter 1 (eENT1) as a crucial regulator of plasma adenosine, suggesting a protective role for erythrocyte ADORA2B-mediated 2,3-BPG production in cases of chronic anemia [217].

The field of metabolomics use in chronic anemia is evolving rapidly, integrating advance techniques as the aforementioned ones. In clinical practice, untargeted metabolomics enables the development of powerful algorithms for disease classification and prediction with dimensional reduction techniques combined with support vector machines, which allow for effective models for small patient cohorts [218]. Metabolomics in chronic anemia may also be helpful in interpreting challenging genomic variants associated with disease prognosis and severity, assisting clinicians and laboratories in understanding complex genetic data [219]. As research advances, standardization across laboratories is expected to improve, mitigating some of these challenges. Additionally, many traditional biochemical assays may eventually be replaced by comprehensive metabolomics-based tests, similar to the evolution in molecular genetic testing over recent decades. This shift toward broader omics-based platforms is promising, given the wide-ranging applicability of metabolomics to contribute to both research and clinical practice.

Future directions include developing liquid biopsy techniques aiming to measure circulating metabolites or exosomes, offering minimally invasive methods to monitor cancer metabolism and treatment responses [220]. Integrating multi-omics data may identify comprehensive metabolic biomarker signatures representing the chronic anemia cell metabolic state, while advances in imaging and biosensor technologies could allow real-time monitor of metabolic changes, facilitating timely therapeutic adjustments [221]. Developing predictive biomarkers to identify patients who would benefit most from specific metabolic targeted therapies will facilitate personalized treatment approaches and patient outcomes [222]. By addressing these challenges, significant progress will be made in effectively targeting metabolism in diseases of chronic anemia in the elderly.

## 6. Conclusions

Diseases of chronic anemia in the elderly are a field of continuous research in order to improve the clinical manifestations and the quality of life in this specific population. Metabolic alterations have been recognized as significant contributors to the pathogenesis and progression of diseases of chronic anemia in older adults, mainly in myelodysplastic syndromes and multiple myeloma, while novel metabolic-targeting therapeutic options have emerged for β-thalassemia, which has been recognized in recent as a chronic disease, characterized by accelerated aging. Further research is needed to deepen our understanding of the complex mechanisms underlying the aforementioned diseases; continued studies are vital for improving our knowledge of metabolic dysregulation in diseases of chronic anemia in the elderly and enhancing novel treatment approaches.

## Figures and Tables

**Table 1:** A summary of medication targeting metabolism in MDSs, categorized by type, metabolite targets, and study model [BMP6: Bone Morphogenetic Protein 6; GLS1: Glutaminase 1; IDH1/2: Isocitrate Dehydrogenase; PKM2: pyruvate kinase M2].

Reference	Medication	Target	Model
[80,81]	3-Bromopyruvate (3-BrPA) (Glycolytic enzyme inhibitor)	Hexokinase	In vivo
[80]	2-Deoxy-D-glucose (2-DG) (Glycolytic enzyme inhibitor)	Hexokinase	In vivo
[84]	Micheliolide (MCL) prodrug (ACT002) (Glycolytic enzyme inhibitor)	PKM2	In vivo
[85]	Telaglenastat (CB-839) (Glutaminase inhibitor)	GLS1	Phase II clinical trial
[87]	Ivosidenib (IDH inhibitor)	IDH1	Phase II clinical trial
[87]	Enasidenib (IDH inhibitor)	IDH2	Phase clinical trial II
[87]	Olaparib [poly-ADP-ribose-polymerase (PARP) inhibitor]	PARP	In vivo
[94]	Rusfertide (PTG-300) (Hepcidin agonist)	Ferroportin	Phase clinical trial II
[94]	Vamifeport (VIT-2763) (Hepcidin agonist)	Ferroportin	Phase II clinical trial
[94]	PR73 (mini-hepcidin) (Hepcidin agonist)	BMP6	In vivo

## Data Availability

The authors confirm that the data supporting the conclusions of this review are found within the article.

## References

[1] Gaskell H., Derry S., Andrew Moore R., McQuay H.J. (2008). Prevalence of Anaemia in Older Persons: Systematic Review. BMC Geriatr..

[2] Blanc B. (1968). Nutritional Anaemias. Report of a WHO Scientific Group. WHO Tech. Rep. Ser..

[3] Shavelle R.M., MacKenzie R., Paculdo D.R. (2012). Anemia and Mortality in Older Persons: Does the Type of Anemia Affect Survival?. Int. J. Hematol..

[4] Stauder R., Valent P., Theurl I. (2018). Anemia at Older Age: Etiologies, Clinical Implications, and Management. Blood.

[5] Schneider A., Jonassaint C., Sharrett A., Mosley T., Astor B., Selvin E., Coresh J., Gottesman R. (2015). Hemoglobin, Anemia, and Cognitive Function: The Atherosclerosis Risk in Communities Study. J. Gerontol. A Biol. Sci. Med. Sci..

[6] Al Saeed M., Ahmed S., Seyadi K., Ahmed A., Alawi S.A., Abulsaad K. (2022). The Prevalence and Impact of Anemia on Hospitalized Older Adults: A Single Center Experience from Bahrain. J. Taibah Univ. Med. Sci..

[7] Oram J.F., Vaughan A.M. (2006). ATP-Binding Cassette Cholesterol Transporters and Cardiovascular Disease. Circ. Res..

[8] Macciò A., Madeddu C. (2012). Management of Anemia of Inflammation in the Elderly. Anemia.

[9] Bruserud Ø., Vo A.K., Rekvam H. (2022). Hematopoiesis, Inflammation and Aging—The Biological Background and Clinical Impact of Anemia and Increased C-Reactive Protein Levels on Elderly Individuals. J. Clin. Med..

[10] Creangă E.-C., Stan R., Nicolae A.-C., Drăgoi C.M., Dumitrescu I.-B. (2025). Personalized Therapeutic Advances in Erythropoietin Signaling: From Anemia Management to Extensive Clinical Applications. Pharmaceutics.

[11] Kondoh H., Kameda M., Yanagida M. (2021). Whole Blood Metabolomics in Aging Research. Int. J. Mol. Sci..

[12] Khanijou J.K., Kulyk H., Bergès C., Khoo L.W., Ng P., Yeo H.C., Helmy M., Bellvert F., Chew W., Selvarajoo K. (2022). Metabolomics and Modelling Approaches for Systems Metabolic Engineering. Metab. Eng. Commun..

[13] Qiu S., Cai Y., Yao H., Lin C., Xie Y., Tang S., Zhang A. (2023). Small Molecule Metabolites: Discovery of Biomarkers and Therapeutic Targets. Signal Transduct. Target. Ther..

[14] Hamany Djande C.Y., Pretorius C., Tugizimana F., Piater L.A., Dubery I.A. (2020). Metabolomics: A Tool for Cultivar Phenotyping and Investigation of Grain Crops. Agronomy.

[15] Tsoukalas D., Sarandi E., Fragoulakis V., Xenidis S., Mhliopoulou M., Charta M., Paramera E., Papakonstantinou E., Tsatsakis A. (2024). Metabolomics-Based Treatment for Chronic Diseases: Results from a Multidisciplinary Clinical Study. BMJ Nutr. Prev. Health.

[16] Arvanitakis K., Chatzikalil E., Kalopitas G., Patoulias D., Popovic D.S., Metallidis S., Kotsa K., Germanidis G., Koufakis T. (2024). Metabolic Dysfunction-Associated Steatotic Liver Disease and Polycystic Ovary Syndrome: A Complex Interplay. J. Clin. Med..

[17] Chatzikalil E., Asvestas D., Tzeis S., Solomou E.E. (2025). Clonal Hematopoiesis of Intermediate Potential in Atrial Fibrillation: A Critical View of Current Knowledge as a Springboard for Future Research. Diagnostics.

[18] Chatzikalil E., Arvanitakis K., Filippatos F., Diamantopoulos P.T., Koufakis T., Solomou E.E. (2025). Diagnostic and Therapeutic Implications of the SUMOylation Pathway in Acute Myeloid Leukemia. Cancers.

[19] Chatzikalil E., Kattamis A., Diamantopoulos P., Solomou E.E. (2023). New-Onset Aplastic Anemia after SARS-CoV-2 Vaccination. Int. J. Hematol..

[20] Chatzikalil E., Roka K., Diamantopoulos P.T., Rigatou E., Avgerinou G., Kattamis A., Solomou E.E. (2024). Venetoclax Combination Treatment of Acute Myeloid Leukemia in Adolescents and Young Adult Patients. J. Clin. Med..

[21] Kaiser L., Weinschrott H., Quint I., Blaess M., Csuk R., Jung M., Kohl M., Deigner H.-P. (2020). Metabolite Patterns in Human Myeloid Hematopoiesis Result from Lineage-Dependent Active Metabolic Pathways. Int. J. Mol. Sci..

[22] Balasubramanian P., Howell P.R., Anderson R.M. (2017). Aging and Caloric Restriction Research: A Biological Perspective With Translational Potential. eBioMedicine.

[23] Shyh-Chang N., Daley G.Q., Cantley L.C. (2013). Stem Cell Metabolism in Tissue Development and Aging. Development.

[24] Ma S., Sun S., Geng L., Song M., Wang W., Ye Y., Ji Q., Zou Z., Wang S.I., He X. (2020). Caloric Restriction Reprograms the Single-Cell Transcriptional Landscape of Rattus Norvegicus Aging. Cell.

[25] Hennrich M.L., Romanov N., Horn P., Jaeger S., Eckstein V., Steeples V., Ye F., Ding X., Poisa-Beiro L., Lai M.C. (2018). Cell-Specific Proteome Analyses of Human Bone Marrow Reveal Molecular Features of Age-Dependent Functional Decline. Nat. Commun..

[26] Comi T.J., Do T.D., Rubakhin S.S., Sweedler J. (2017). V Categorizing Cells on the Basis of Their Chemical Profiles: Progress in Single-Cell Mass Spectrometry. J. Am. Chem. Soc..

[27] Li S., Vazquez J.M., Sudmant P.H. (2023). The Evolution of Aging and Lifespan. Trends Genet..

[28] Lokhov P.G., Balashova E.E., Maslov D.L., Trifonova O.P., Archakov A.I. (2024). Aging and Pathological Conditions Similarity Revealed by Meta-Analysis of Metabolomics Studies Suggests the Existence of the Health and Age-Related Metapathway. Metabolites.

[29] Fang W., Chen S., Jin X., Liu S., Cao X., Liu B. (2023). Metabolomics in Aging Research: Aging Markers from Organs. Front. Cell Dev. Biol..

[30] Madu A.J., Ughasoro M.D. (2016). Anaemia of Chronic Disease: An In-Depth Review. Med. Princ. Pract..

[31] Garcia-Manero G. (2023). Myelodysplastic Syndromes: 2023 Update on Diagnosis, Risk-Stratification, and Management. Am. J. Hematol..

[32] Sanchez-Villalobos M., Blanquer Blanquer M., Moraleda J., Salido E., Perez-Oliva A. (2022). New Insights Into Pathophysiology of β-Thalassemia. Front. Med..

[33] Sadiq I.Z., Abubakar F.S., Usman H.S., Abdullahi A.D., Ibrahim B., Kastayal B.S., Ibrahim M., Hassan H.A. (2024). Thalassemia: Pathophysiology, Diagnosis, and Advances in Treatment. Thalass. Rep..

[34] Sekeres M., Taylor J. (2022). Diagnosis and Treatment of Myelodysplastic Syndromes: A Review. JAMA.

[35] Rodriguez-Sevilla J.J., Adema V., Garcia-Manero G., Colla S. (2023). Emerging Treatments for Myelodysplastic Syndromes: Biological Rationales and Clinical Translation. Cell Rep. Med..

[36] Li Z., Zhang H. (2016). Reprogramming of Glucose, Fatty Acid and Amino Acid Metabolism for Cancer Progression. Cell. Mol. Life Sci..

[37] Chung S.S., Park C.Y. (2017). Aging, Hematopoiesis, and the Myelodysplastic Syndromes. Hematology.

[38] Kokkaliaris K.D., Scadden D.T. (2020). Cell Interactions in the Bone Marrow Microenvironment Affecting Myeloid Malignancies. Blood Adv..

[39] Nakamura-Ishizu A., Ito K., Suda T. (2020). Hematopoietic Stem Cell Metabolism during Development and Aging. Dev. Cell.

[40] Suda T., Takubo K., Semenza G. (2011). Metabolic Regulation of Hematopoietic Stem Cells in the Hypoxic Niche. Cell Stem Cell.

[41] Takubo K., Goda N., Yamada W., Iriuchishima H., Ikeda E., Kubota Y., Shima H., Johnson R.S., Hirao A., Suematsu M. (2010). Regulation of the HIF-1α Level Is Essential for Hematopoietic Stem Cells. Cell Stem Cell.

[42] Pronk E., Raaijmakers M.H.G.P. (2019). The Mesenchymal Niche in MDS. Blood.

[43] Liu J., Gao J., Liang Z., Gao C., Niu Q., Wu F., Zhang L. (2022). Mesenchymal Stem Cells and Their Microenvironment. Stem Cell Res. Ther..

[44] Goulard M., Dosquet C., Bonnet D. (2018). Role of the Microenvironment in Myeloid Malignancies. Cell. Mol. Life Sci..

[45] Zhou S., Chen S., Jiang Q., Pei M. (2019). Determinants of Stem Cell Lineage Differentiation toward Chondrogenesis versus Adipogenesis. Cell. Mol. Life Sci..

[46] Grayson W., Zhao F., Bunnell B., Ma T. (2007). Hypoxia Enhances Proliferation and Tissue Formation of Human Mesenchymal Stem Cell. Biochem. Biophys. Res. Commun..

[47] Liberti M.V., Locasale J.W. (2016). The Warburg Effect: How Does It Benefit Cancer Cells?. Trends Biochem. Sci..

[48] Pattappa G., Heywood H., de Bruijn J., Lee D. (2011). The Metabolism of Human Mesenchymal Stem Cells During Proliferation and Differentiation. J. Cell. Physiol..

[49] Stevens B., Engel K., Gillen A., Culp-Hill R., D’Alessandro A., Alper S., Pollyea D., Jordan C. (2021). Unique Metabolic Vulnerabilities of Myelodysplastic Syndrome Stem Cells. Blood.

[50] Jones C.L., Inguva A., Jordan C.T. (2021). Targeting Energy Metabolism in Cancer Stem Cells: Progress and Challenges in Leukemia and Solid Tumors. Cell Stem Cell.

[51] Patel S.B., Nemkov T., D’Alessandro A., Welner R.S. (2022). Deciphering Metabolic Adaptability of Leukemic Stem Cells. Front. Oncol..

[52] Richardson C., Yan S., Vestal C.G. (2015). Oxidative Stress, Bone Marrow Failure, and Genome Instability in Hematopoietic Stem Cells. Int. J. Mol. Sci..

[53] Poulaki A., Katsila T., Stergiou I.E., Giannouli S., Gόmez-Tamayo J.C., Piperaki E.-T., Kambas K., Dimitrakopoulou A., Patrinos G.P., Tzioufas A.G. (2020). Bioenergetic Profiling of the Differentiating Human MDS Myeloid Lineage with Low and High Bone Marrow Blast Counts. Cancers.

[54] Gong T., Zheng C., Ou X., Zheng J., Yu J., Chen S., Duan Y., Liu W. (2022). Glutamine Metabolism in Cancers: Targeting the Oxidative Homeostasis. Front. Oncol..

[55] Yoo H.C., Yu Y.C., Sung Y., Han J.M. (2020). Glutamine Reliance in Cell Metabolism. Exp. Mol. Med..

[56] Matre P., Velez Lujan J., Jacamo R., Qi Y., Su X., Cai T., Chan S., Lodi A., Sweeney S., Ma H. (2016). Inhibiting Glutaminase in Acute Myeloid Leukemia: Metabolic Dependency of Selected AML Subtypes. Oncotarget.

[57] Modoux M., Rolhion N., Mani S., Sokol H. (2020). Tryptophan Metabolism as a Pharmacological Target. Trends Pharmacol. Sci..

[58] Lee G.K., Park H., MacLeod M., Chandler P., Munn D., Mellor A. (2003). Tryptophan Deprivation Sensitizes Activated T Cells to Apoptosis Prior to Cell Division. Immunology.

[59] Giannattasio S., Dri M., Merra G., Caparello G., Rampello T., Renzo L. (2021). Effects of Fatty Acids on Hematological Neoplasms: A Mini Review. Nutr. Cancer.

[60] Carracedo A., Cantley L.C., Pandolfi P.P. (2013). Cancer Metabolism: Fatty Acid Oxidation in the Limelight. Nat. Rev. Cancer.

[61] Dang L., Yen K., Attar E. (2016). IDH Mutations in Cancer and Progress toward Development of Targeted Therapeutics. Ann. Oncol..

[62] Kirkman H., Rolfo M., Ferraris A., Gaetani G. (1999). Mechanisms of Protection of Catalase by NADPH. J. Biol. Chem..

[63] Chang L.-C., Chiang S.-K., Chen S.-E., Hung M.-C. (2022). Targeting 2-Oxoglutarate Dehydrogenase for Cancer Treatment. Am. J. Cancer Res..

[64] Lin C.-C., Hou H.-A., Chou W.-C., Kuo Y.-Y., Liu C.-Y., Chen C.-Y., Lai Y.-J., Tseng M.-H., Huang C.-F., Chiang Y.-C. (2014). IDH Mutations Are Closely Associated with Mutations of DNMT3A, ASXL1 and SRSF2 in Patients with Myelodysplastic Syndromes and Are Stable during Disease Evolution. Am. J. Hematol..

[65] Pirozzi C., Yan H. (2021). The Implications of *IDH* Mutations for Cancer Development and Therapy. Nat. Rev. Clin. Oncol..

[66] Komrokji R., Al Ali N., Chan O., Sweet K., Kuykendall A., Lancet J., Padron E., Sallman D. (2022). *IDH* Mutations Are Enriched in Myelodysplastic Syndrome Patients with Severe Neutropenia and Can Be a Potential for Targeted Therapy. Haematologica.

[67] Sebastiani G., Pantopoulos K. (2011). Disorders Associated with Systemic or Local Iron Overload: From Pathophysiology to Clinical Practice. Metallomics.

[68] Chatzikalil E., Arvanitakis K., Kalopitas G., Florentin M., Germanidis G., Koufakis T., Solomou E.E. (2025). Hepatic Iron Overload and Hepatocellular Carcinoma: New Insights into Pathophysiological Mechanisms and Therapeutic Approaches. Cancers.

[69] Słomka A., Pokrzywa A., Strzała D., Kubiaczyk M., Wesołowska O., Denkiewicz K., Styczynski J. (2024). The Role of Hepcidin in Myelodysplastic Syndromes (MDS): A Systematic Review of Observational Studies. Cancers.

[70] Kohgo Y., Ikuta K., Ohtake T., Torimoto Y., Kato J. (2008). Body Iron Metabolism and Pathophysiology of Iron Overload. Int. J. Hematol..

[71] Knutson M., Wessling-Resnick M. (2003). Iron Metabolism in the Reticuloendothelial System. Crit. Rev. Biochem. Mol. Biol..

[72] Frascatani R., Colella M., Monteleone G. (2024). Hepcidin Is a Valuable Therapeutic Target for Colorectal Cancer. Cancers.

[73] Ru Q., Li Y., Chen L., Wu Y., Min J., Wang F. (2024). Iron Homeostasis and Ferroptosis in Human Diseases: Mechanisms and Therapeutic Prospects. Signal Transduct. Target. Ther..

[74] Patnaik M.M., Tefferi A. (2021). Myelodysplastic Syndromes with Ring Sideroblasts (MDS-RS) and MDS/Myeloproliferative Neoplasm with RS and Thrombocytosis (MDS/MPN-RS-T)—“2021 Update on Diagnosis, Risk-Stratification, and Management”. Am. J. Hematol..

[75] Stoian I., Manolescu B., Atanasiu V., Lupescu O., Busu C. (2007). IL-6-STAT-3-Hepcidin: Linking Inflammation to the Iron Metabolism. Rom. J. Intern. Med..

[76] Coffey R., Jung G., Olivera J.D., Karin G., Pereira R.C., Nemeth E., Ganz T. (2022). Erythroid Overproduction of Erythroferrone Causes Iron Overload and Developmental Abnormalities in Mice. Blood.

[77] Ambaglio I., Malcovati L., Papaemmanuil E., Laarakkers C., Porta M., Gallì A., Da Vià M., Bono E., Ubezio M., Travaglino E. (2013). Inappropriately Low Hepcidin Levels in Patients with Myelodysplastic Syndrome Carrying a Somatic Mutation of SF3B1. Haematologica.

[78] Camaschella C., Nai A., Silvestri L. (2020). Iron Metabolism and Iron Disorders Revisited in the Hepcidin Era. Haematologica.

[79] Seiler K., Humbert M., Minder P., Mashimo I., Schläfli A., Krauer-Shan D., Federzoni E., Vu B., Moresco J., Yates J. (2022). Hexokinase 3 Enhances Myeloid Cell Survival via Non-Glycolytic Functions. Cell Death Dis..

[80] Sheng H. (2016). Glycolysis Inhibitors for Anticancer Therapy: A Review of Recent Patents. Recent Pat. Anticancer. Drug Discov..

[81] Zhou R.-Q., Wang X., Ye Y.-B., Lu B., Wang J., Guo Z.-W., Mo W.-J., Yang Z., Srisuk P., Yan L.-P. (2022). Prevention of Acute Graft-vs.-host Disease by Targeting Glycolysis and MTOR Pathways in Activated T Cells. Exp. Ther. Med..

[82] Xia L., Jiang Y., Zhang X.-H., Wang X.-R., Wei R., Qin K., Lu Y. (2021). SUMOylation Disassembles the Tetrameric Pyruvate Kinase M2 to Block Myeloid Differentiation of Leukemia Cells. Cell Death Dis..

[83] Wang L., Yang L., Yang Z., Tang Y., Tao Y., Zhan Q., Lei L., Jing Y., Jiang X., Jin H. (2019). Glycolytic Enzyme PKM2 Mediates Autophagic Activation to Promote Cell Survival in NPM1-Mutated Leukemia. Int. J. Biol. Sci..

[84] Li J., Li S., Guo J., Li Q., Long J., Ma C., Ding Y., Yan C., Li L., Wu Z. (2018). Natural Product Micheliolide (MCL) Irreversibly Activates Pyruvate Kinase M2 and Suppresses Leukemia. J. Med. Chem..

[85] DiNardo C., Verma D., Baran N., Bhagat T., Skwarska A., Lodi A., Saxena K., Cai T., Su X., Guerra V. (2024). Glutaminase Inhibition in Combination with Azacytidine in Myelodysplastic Syndromes: A Phase 1b/2 Clinical Trial and Correlative Analyses. Nat. Cancer.

[86] Testa U., Castelli G., Pelosi E. (2020). Isocitrate Dehydrogenase Mutations in Myelodysplastic Syndromes and in Acute Myeloid Leukemias. Cancers.

[87] Gbyli R., Song Y., Liu W., Gao Y., Biancon G., Chandhok N., Wang X., Fu X., Patel A., Sundaram R. (2022). In Vivo Anti-Tumor Effect of PARP Inhibition in IDH1/2 Mutant MDS/AML Resistant to Targeted Inhibitors of Mutant IDH1/2. Leukemia.

[88] Sebert M., Cluzeau T., Rauzy O.B., Bastard A.S., Dimicoli-Salazar S., Thepot S., Peterlin P., Park S., Gourin M.-P., Brehar O. (2021). Ivosidenib Monotherapy Is Effective in Patients with IDH1 Mutated Myelodysplastic Syndrome (MDS): The Idiome Phase 2 Study by the GFM Group. Blood.

[89] Stempel J.M., Kewan T., Zeidan A.M. (2025). Advances and Challenges in the Management of Myelodysplastic Syndromes. Cancers.

[90] Stein E.M., Fathi A.T., DiNardo C.D., Pollyea D.A., Roboz G.J., Collins R., Sekeres M.A., Stone R.M., Attar E.C., Frattini M.G. (2020). Enasidenib in Patients with Mutant IDH2 Myelodysplastic Syndromes: A Phase 1 Subgroup Analysis of the Multicentre, AG221-C-001 Trial. Lancet Haematol..

[91] DiNardo C.D., Venugopal S., Lachowiez C., Takahashi K., Loghavi S., Montalban-Bravo G., Wang X., Carraway H., Sekeres M., Sukkur A. (2023). Targeted Therapy with the Mutant IDH2 Inhibitor Enasidenib for High-Risk IDH2-Mutant Myelodysplastic Syndrome. Blood Adv..

[92] Leitch H., Leger C., Goodman T., Wong K., Wong D., Ramadan K., Rollins M., Barnett M., Galbraith P., Vickars L. (2008). Improved Survival in Patients with Myelodysplastic Syndrome Receiving Iron Chelation Therapy. Clin. Leuk..

[93] DivakarJose R.R., Delhikumar C.G., Ram Kumar G. (2020). Efficacy and Safety of Combined Oral Chelation with Deferiprone and Deferasirox on Iron Overload in Transfusion Dependent Children with Thalassemia—A Prospective Observational Study. Indian J. Pediatr..

[94] Sandnes M., Reikvam H. (2024). Hepcidin as a Therapeutic Target in Iron Overload. Expert Opin. Ther. Targets.

[95] Li X., Zou C., Xiang X., Zhao L., Chen M., Yang C., Wu Y. (2025). Myelodysplastic Neoplasms (MDS): Pathogenesis and Therapeutic Prospects. Biomolecules.

[96] Oliva E.N., Huey K., Deshpande S., Turner M., Chitnis M., Schiller E., Tang D., Yucel A., Hughes C., Shah F. (2022). A Systematic Literature Review of the Relationship between Serum Ferritin and Outcomes in Myelodysplastic Syndromes. J. Clin. Med..

[97] Durairaj S., Chew S., Hyslop A., Keenan N., Groves M.J., Tauro S. (2011). Predicted Costs of Iron-chelators in Myelodysplastic Syndromes: A 10-year Analysis Based on Actual Prevalence and Red Cell Transfusion Rates. Am. J. Hematol..

[98] Efficace F., Santini V., La Nasa G., Cottone F., Finelli C., Borin L., Quaresmini G., Di Tucci A.A., Volpe A., Cilloni D. (2016). Health-Related Quality of Life in Transfusion-Dependent Patients with Myelodysplastic Syndromes: A Prospective Study to Assess the Impact of Iron Chelation Therapy. BMJ Support. Palliat. Care.

[99] Malard F., Neri P., Bahlis N.J., Terpos E., Moukalled N., Hungria V.T.M., Manier S., Mohty M. (2024). Multiple Myeloma. Nat. Rev. Dis. Prim..

[100] Utley A., Lipchick B., Lee K.P., Nikiforov M.A. (2020). Targeting Multiple Myeloma through the Biology of Long-Lived Plasma Cells. Cancers.

[101] Forster S., Radpour R., Ochsenbein A.F. (2023). Molecular and Immunological Mechanisms of Clonal Evolution in Multiple Myeloma. Front. Immunol..

[102] Yang P., Qu Y., Wang M., Chu B., Chen W., Zheng Y., Niu T., Qian Z. (2022). Pathogenesis and Treatment of Multiple Myeloma. MedComm.

[103] Lu Q., Yang D., Li H., Niu T., Tong A. (2024). Multiple Myeloma: Signaling Pathways and Targeted Therapy. Mol. Biomed..

[104] Facon T., Leleu X., Manier S. (2024). How I Treat Multiple Myeloma in Geriatric Patients. Blood.

[105] Dima D., Jiang D., Singh D.J., Hasipek M., Shah H.S., Ullah F., Khouri J., Maciejewski J.P., Jha B.K. (2022). Multiple Myeloma Therapy: Emerging Trends and Challenges. Cancers.

[106] Ito S. (2020). Proteasome Inhibitors for the Treatment of Multiple Myeloma. Cancers.

[107] Zhou X., He R., Hu W.-X., Luo S., Hu J. (2024). Targeting Myeloma Metabolism: How Abnormal Metabolism Contributes to Multiple Myeloma Progression and Resistance to Proteasome Inhibitors. Neoplasia.

[108] Liu H., Wang S., Wang J., Guo X., Song Y., Fu K., Gao Z., Liu D., He W., Yang L.-L. (2025). Energy Metabolism in Health and Diseases. Signal Transduct. Target. Ther..

[109] Cisewski S.E., Zhang L., Kuo J., Wright G.J., Wu Y., Kern M.J., Yao H. (2015). The Effects of Oxygen Level and Glucose Concentration on the Metabolism of Porcine TMJ Disc Cells. Osteoarthr. Cartil..

[110] Drochioiu G. (2023). Multifactorial Distress, the Warburg Effect, and Respiratory and PH Imbalance in Cancer Development. Stresses.

[111] Revheim M.-E., Stokke C., Nørgaard J.N., Phillips H.F., Sherwani A.G., Schjesvold F., Connelly J.P. (2021). New Targets for PET Imaging of Myeloma. Hemato.

[112] Xu S., Zhou T., Doh H.M., Trinh K.R., Catapang A., Lee J.T., Braas D., Bayley N.A., Yamada R.E., Vasuthasawat A. (2019). An HK2 Antisense Oligonucleotide Induces Synthetic Lethality in HK1−HK2+ Multiple Myeloma. Cancer Res..

[113] Wang X., Shen X., Yan Y., Li H. (2021). Pyruvate Dehydrogenase Kinases (PDKs): An Overview toward Clinical Applications. Biosci. Rep..

[114] Tataranni T., Agriesti F., Pacelli C., Ruggieri V., Laurenzana I., Mazzoccoli C., Della Sala G., Panebianco C., Pazienza V., Capitanio N. (2019). Dichloroacetate Affects Mitochondrial Function and Stemness-Associated Properties in Pancreatic Cancer Cell Lines. Cells.

[115] Roh J.-L., Park J.Y., Kim E.H., Jang H.J., Kwon M. (2016). Activation of Mitochondrial Oxidation by PDK2 Inhibition Reverses Cisplatin Resistance in Head and Neck Cancer. Cancer Lett..

[116] Barba I., Carrillo-Bosch L., Seoane J. (2024). Targeting the Warburg Effect in Cancer: Where Do We Stand?. Int. J. Mol. Sci..

[117] Arponen M., Jalava N., Widjaja N., Ivaska K.K. (2022). Glucose Transporters GLUT1, GLUT3, and GLUT4 Have Different Effects on Osteoblast Proliferation and Metabolism. Front. Physiol..

[118] Dalva-Aydemir S., Bajpai R., Martinez M., Adekola K.U.A., Kandela I., Wei C., Singhal S., Koblinski J.E., Raje N.S., Rosen S.T. (2015). Targeting the Metabolic Plasticity of Multiple Myeloma with FDA-Approved Ritonavir and Metformin. Clin. Cancer Res..

[119] Abdollahi P., Vandsemb E.N., Elsaadi S., Røst L.M., Yang R., Hjort M.A., Andreassen T., Misund K., Slørdahl T.S., Rø T.B. (2021). Phosphatase of Regenerating Liver-3 Regulates Cancer Cell Metabolism in Multiple Myeloma. FASEB J..

[120] Tan X.-Q., Guo A.-Y., Zheng L.-F., Xiong J. (2025). Research Progress on FOXM1 in Ovarian Cancer Diagnosis and Therapeutics. Front. Oncol..

[121] Singh M.K., Han S., Kim S., Kang I. (2024). Targeting Lipid Metabolism in Cancer Stem Cells for Anticancer Treatment. Int. J. Mol. Sci..

[122] Arvanitakis K., Chatzikalil E., Antza C., Topalidis C., Kalopitas G., Solomou E., Kotsis V., Germanidis G., Koufakis T., Doumas M. (2025). Pediatric Familial Hypercholesterolemia: Targeting Intestinal Absorption and Other Therapeutic Strategies. Nutrients.

[123] Butler L.M., Perone Y., Dehairs J., Lupien L.E., de Laat V., Talebi A., Loda M., Kinlaw W.B., Swinnen J.V. (2020). Lipids and Cancer: Emerging Roles in Pathogenesis, Diagnosis and Therapeutic Intervention. Adv. Drug Deliv. Rev..

[124] Duan Y., Gong K., Xu S., Zhang F., Meng X., Han J. (2022). Regulation of Cholesterol Homeostasis in Health and Diseases: From Mechanisms to Targeted Therapeutics. Signal Transduct. Target. Ther..

[125] Cuyàs E., Pedarra S., Verdura S., Pardo M.A., Espin Garcia R., Serrano-Hervás E., Llop-Hernández À., Teixidor E., Bosch-Barrera J., López-Bonet E. (2024). Fatty Acid Synthase (FASN) Is a Tumor-Cell-Intrinsic Metabolic Checkpoint Restricting T-Cell Immunity. Cell Death Discov..

[126] Chen M., Huang J. (2019). The Expanded Role of Fatty Acid Metabolism in Cancer: New Aspects and Targets. Precis. Clin. Med..

[127] Liu Q., Luo Q., Halim A., Song G. (2017). Targeting Lipid Metabolism of Cancer Cells: A Promising Therapeutic Strategy for Cancer. Cancer Lett..

[128] Delmas D., Cotte A.K., Connat J.-L., Hermetet F., Bouyer F., Aires V. (2023). Emergence of Lipid Droplets in the Mechanisms of Carcinogenesis and Therapeutic Responses. Cancers.

[129] Diedrich J.D., Herroon M.K., Rajagurubandara E., Podgorski I. (2018). The Lipid Side of Bone Marrow Adipocytes: How Tumor Cells Adapt and Survive in Bone. Curr. Osteoporos. Rep..

[130] Li J., Zhao J., Wang S., Wu R., Duan S., Wang H. (2025). A Case Report of IgA-κ Type Multiple Myeloma Complicated With Hyperlipidemia. Clin. Case Rep..

[131] Tirado-Vélez J.M., Benítez-Rondán A., Cózar-Castellano I., Medina F., Perdomo G. (2012). Low-Density Lipoprotein Cholesterol Suppresses Apoptosis in Human Multiple Myeloma Cells. Ann. Hematol..

[132] Morofuji Y., Nakagawa S., Ujifuku K., Fujimoto T., Otsuka K., Niwa M., Tsutsumi K. (2022). Beyond Lipid-Lowering: Effects of Statins on Cardiovascular and Cerebrovascular Diseases and Cancer. Pharmaceuticals.

[133] Xiao M., Xu J., Wang W., Zhang B., Liu J., Li J., Xu H., Zhao Y., Yu X., Shi S. (2023). Functional Significance of Cholesterol Metabolism in Cancer: From Threat to Treatment. Exp. Mol. Med..

[134] Mollinedo F., Iglesia-Vicente J., Gajate C., Estella-Hermoso de Mendoza A., Villa-Pulgarin J., Campanero M., Blanco-Prieto M. (2010). Lipid Raft-Targeted Therapy in Multiple Myeloma. Oncogene.

[135] Li Y.C., Park M.J., Ye S.-K., Kim C.-W., Kim Y.-N. (2006). Elevated Levels of Cholesterol-Rich Lipid Rafts in Cancer Cells Are Correlated with Apoptosis Sensitivity Induced by Cholesterol-Depleting Agents. Am. J. Pathol..

[136] Zheng X., Huang Z., Liu Z., Zheng Z., Zhang Y., Aweya J.J. (2022). Transcriptome Analysis Reveals That SREBP Modulates a Large Repertoire of Genes Involved in Key Cellular Functions in Penaeus Vannamei, Although the Majority of the Dysregulated Genes Are Unannotated. Genes.

[137] Bessot A., Gunter J., McGovern J., Bock N. (2025). Bone Marrow Adipocytes in Cancer: Mechanisms, Models, and Therapeutic Implications. Biomaterials.

[138] Mastrogeorgiou M., Chatzikalil E., Theocharis S., Papoudou-Bai A., Péoc’h M., Mobarki M., Karpathiou G. (2024). The Immune Microenvironment of Cancer of the Uterine Cervix. Histol. Histopathol..

[139] Li Y., Wang L., Wang J., Xin Y., Lyu X. (2024). Relationship between Adipocytes and Hematological Tumors in the Bone Marrow Microenvironment: A Literature Review. Transl. Cancer Res..

[140] Baldelli S., Aiello G., Mansilla Di Martino E., Campaci D., Muthanna F.M.S., Lombardo M. (2024). The Role of Adipose Tissue and Nutrition in the Regulation of Adiponectin. Nutrients.

[141] Panaroni C., Fulzele K., Mori T., Siu K.T., Onyewadume C., Maebius A., Raje N. (2022). Multiple Myeloma Cells Induce Lipolysis in Adipocytes and Uptake Fatty Acids through Fatty Acid Transporter Proteins. Blood.

[142] Fairfield H., Dudakovic A., Khatib C.M., Farrell M., Costa S., Falank C., Hinge M., Murphy C.S., DeMambro V., Pettitt J.A. (2021). Myeloma-Modified Adipocytes Exhibit Metabolic Dysfunction and a Senescence-Associated Secretory Phenotype. Cancer Res..

[143] Sherman C., Peterson S., Frishman W. (2010). Apolipoprotein A-I Mimetic Peptides: A Potential New Therapy for the Prevention of Atherosclerosis. Cardiol. Rev..

[144] Ling Z.-N., Jiang Y.-F., Ru J.-N., Lu J.-H., Ding B., Wu J. (2023). Amino Acid Metabolism in Health and Disease. Signal Transduct. Target. Ther..

[145] Averous J., Lambert-Langlais S., Mesclon F., Carraro V., Parry L., Jousse C., Bruhat A., Maurin A.-C., Pierre P., Proud C.G. (2016). GCN2 Contributes to MTORC1 Inhibition by Leucine Deprivation through an ATF4 Independent Mechanism. Sci. Rep..

[146] Soncini D., Minetto P., Martinuzzi C., Becherini P., Fenu V., Guolo F., Todoerti K., Calice G., Contini P., Miglino M. (2020). Amino Acid Depletion Triggered by ʟ-Asparaginase Sensitizes MM Cells to Carfilzomib by Inducing Mitochondria ROS-Mediated Cell Death. Blood Adv..

[147] Plano F., Gigliotta E., Corsale A.M., Azgomi M.S., Santonocito C., Ingrascì M., Di Carlo L., Augello A.E., Speciale M., Vullo C. (2023). Ferritin Metabolism Reflects Multiple Myeloma Microenvironment and Predicts Patient Outcome. Int. J. Mol. Sci..

[148] Shaw J., Chakraborty A., Nag A., Chattopadyay A., Dasgupta A.K., Bhattacharyya M. (2017). Intracellular Iron Overload Leading to DNA Damage of Lymphocytes and Immune Dysfunction in Thalassemia Major Patients. Eur. J. Haematol..

[149] Yang F., Wu Z., Dai D., Zhang L., Zhang X., Zhang X., Xu Y. (2021). The Iron Chelator Deferoxamine Decreases Myeloma Cell Survival. J. Int. Med. Res..

[150] Strasser-Weippl K., Ludwig H. (2014). Ferritin as Prognostic Marker in Multiple Myeloma Patients Undergoing Autologous Transplantation. Leuk. Lymphoma.

[151] Naeimi Kararoudi M., Nagai Y., Elmas E., de Souza Fernandes Pereira M., Ali S.A., Imus P.H., Wethington D., Borrello I.M., Lee D.A., Ghiaur G. (2020). CD38 Deletion of Human Primary NK Cells Eliminates Daratumumab-Induced Fratricide and Boosts Their Effector Activity. Blood J. Am. Soc. Hematol..

[152] Viola D., Dona A., Caserta E., Troadec E., Besi F., McDonald T., Ghoda L., Gunes E.G., Sanchez J.F., Khalife J. (2021). Daratumumab Induces Mechanisms of Immune Activation through CD38+ NK Cell Targeting. Leukemia.

[153] Pinto V.M., Forni G.L. (2020). Management of Iron Overload in Beta-Thalassemia Patients: Clinical Practice Update Based on Case Series. Int. J. Mol. Sci..

[154] Delaporta P., Chatzikalil E., Ladis V., Moraki M., Kattamis A. (2023). Evolving Changes in the Characteristics of Death in Transfusion Dependent Thalassemia in Greece. Blood.

[155] Delaporta P., Chatzikalil E., Kyriakopoulou D., Berdalli S., Chouliara V., Hatzieleftheriou M.-I., Mylona S., Kattamis A. (2024). Abrupt Increases in Ferritin Levels May Indicate a Malignant Process and Not Changes in Iron Overload in Thalassemic Patients. Blood.

[156] Zhang D.-L., Ghosh M.C., Rouault T.A. (2014). The Physiological Functions of Iron Regulatory Proteins in Iron Homeostasis—An Update. Front. Pharmacol..

[157] Duca L., Di Pierro E., Scaramellini N., Granata F., Graziadei G. (2025). The Relationship Between Non-Transferrin-Bound Iron (NTBI), Labile Plasma Iron (LPI), and Iron Toxicity. Int. J. Mol. Sci..

[158] Nairz M., Theurl I., Swirski F.K., Weiss G. (2017). “Pumping Iron”—How Macrophages Handle Iron at the Systemic, Microenvironmental, and Cellular Levels. Pflügers Arch.-Eur. J. Physiol..

[159] Voskou S., Aslan M., Fanis P., Phylactides M., Kleanthous M. (2015). Oxidative Stress in β-Thalassaemia and Sickle Cell Disease. Redox Biol..

[160] Rivadeneira D.B., Thosar S., Quann K., Gunn W.G., Dean V.G., Xie B., Parise A., McGovern A.C., Spahr K., Lontos K. (2025). Oxidative-Stress-Induced Telomere Instability Drives T Cell Dysfunction in Cancer. Immunity.

[161] Gluba-Brzózka A., Franczyk B., Rysz-Górzyńska M., Rokicki R., Koziarska-Rościszewska M., Rysz J. (2021). Pathomechanisms of Immunological Disturbances in β-Thalassemia. Int. J. Mol. Sci..

[162] Alum E.U., Izah S.C., Uti D.E., Ugwu O.P., Betiang P.A., Basajja M., Ejemot-Nwadiaro R.I. (2025). Targeting Cellular Senescence for Healthy Aging: Advances in Senolytics and Senomorphics. Drug Des. Devel. Ther..

[163] Martyshkina Y.S., Tereshchenko V.P., Bogdanova D.A., Rybtsov S.A. (2023). Reliable Hallmarks and Biomarkers of Senescent Lymphocytes. Int. J. Mol. Sci..

[164] Musharraf S.G., Iqbal A., Ansari S.H., Parveen S., Khan I.A., Siddiqui A.J. (2017). β-Thalassemia Patients Revealed a Significant Change of Untargeted Metabolites in Comparison to Healthy Individuals. Sci. Rep..

[165] El Azab E.F., Abdulmalek S. (2022). Amelioration of Age-Related Multiple Neuronal Impairments and Inflammation in High-Fat Diet-Fed Rats: The Prospective Multitargets of Geraniol. Oxid. Med. Cell. Longev..

[166] Ceja-Galicia Z.A., Cespedes-Acuña C.L.A., El-Hafidi M. (2025). Protection Strategies Against Palmitic Acid-Induced Lipotoxicity in Metabolic Syndrome and Related Diseases. Int. J. Mol. Sci..

[167] Biggelaar L.J.C.J.d., Eussen S.J.P.M., Sep S.J.S., Mari A., Ferrannini E., Dongen M.C.J.M.v., Denissen K.F.M., Wijckmans N.E.G., Schram M.T., Kallen C.J.v.d. (2017). Associations of Dietary Glucose, Fructose, and Sucrose with β-Cell Function, Insulin Sensitivity, and Type 2 Diabetes in the Maastricht Study. Nutrients.

[168] Cockcroft S. (2021). Mammalian Lipids: Structure, Synthesis and Function. Essays Biochem..

[169] Carta G., Murru E., Banni S., Manca C. (2017). Palmitic Acid: Physiological Role, Metabolism and Nutritional Implications. Front. Physiol..

[170] Alruwaili N., Alshdayed F. (2023). Fructose Metabolism and Its Effect on Glucose-Galactose Malabsorption Patients: A Literature Review. Diagnostics.

[171] Le Calvé S., Somme D., Prud’homm J., Corvol A. (2017). Blood Transfusion in Elderly Patients with Chronic Anemia: A Qualitative Analysis of the General Practitioners’ Attitudes. BMC Fam. Pract..

[172] Raffa M., Atig F., Mhalla A., Kerkeni A., Mechri A. (2011). Decreased Glutathione Levels and Impaired Antioxidant Enzyme Activities in Drug-Naive First-Episode Schizophrenic Patients. BMC Psychiatry.

[173] de Wolski K., Fu X., Dumont L.J., Roback J.D., Waterman H., Odem-Davis K., Howie H.L., Zimring J.C. (2016). Metabolic Pathways That Correlate with Post-Transfusion Circulation of Stored Murine Red Blood Cells. Haematologica.

[174] Jiang H.J., Underwood T.C., Bell J.G., Ranjan S., Sasselov D., Whitesides G.M. (2017). Mimicking Lighting-Induced Electrochemistry on the Early Earth. Proc. Natl. Acad. Sci. USA.

[175] Biswas S., Smrity S.Z., Bhuia M.S., Sonia F.A., Aktar M.A., Chowdhury R., Islam T., Islam M.T., Gonçalves Alencar G., Paulo C.L.R. (2024). Beta-Thalassemia: A Pharmacological Drug-Based Treatment. Drugs Drug Candidates.

[176] Iqbal A., Ansari S.H., Parveen S., Khan I.A., Siddiqui A.J., Musharraf S.G. (2018). Hydroxyurea Treated β-Thalassemia Children Demonstrate a Shift in Metabolism Towards Healthy Pattern. Sci. Rep..

[177] Szebeni J., Esketson C., Sampliner R., Hartmann B., Griffin J., Dormandy T., Watson R.R. (1986). Plasma Fatty Acid Pattern Including Diene-conjugated Linoleic Acid in Ethanol Users and Patients with Ethanol-related Liver Disease. Alcohol. Clin. Exp. Res..

[178] Wang S., Ma A., Song S., Quan Q., Zhao X., Zheng X. (2008). Fasting Serum Free Fatty Acid Composition, Waist/Hip Ratio and Insulin Activity in Essential Hypertensive Patients. Hypertens. Res..

[179] Chen Y., Ma Z., Min L., Li H., Wang B., Zhong J., Dai L. (2015). Biomarker Identification and Pathway Analysis by Serum Metabolomics of Lung Cancer. Biomed. Res. Int..

[180] Jackson K.H., Harris W.S., Belury M.A., Kris-Etherton P.M., Calder P.C. (2024). Beneficial Effects of Linoleic Acid on Cardiometabolic Health: An Update. Lipids Health Dis..

[181] Harris W., Pottala J., Varvel S., Borowski J., Ward J., McConnell J. (2013). Erythrocyte Omega-3 Fatty Acids Increase and Linoleic Acid Decreases with Age: Observations from 160,000 Patients. Prostaglandins. Leukot. Essent. Fat. Acids.

[182] Botta A., Forest A., Daneault C., Pantopoulos K., Tantiworawit A., Phrommintikul A., Chattipakorn S., Chattipakorn N., Des Rosiers C., Sweeney G. (2021). Identification of Circulating Endocan-1 and Ether Phospholipids as Biomarkers for Complications in Thalassemia Patients. Metabolites.

[183] Srinoun K., Sathirapongsasuti N., Paiboonsukwong K., Sretrirutchai S., Wongchanchailert M., Fucharoen S. (2019). MiR-144 Regulates Oxidative Stress Tolerance of Thalassemic Erythroid Cell via Targeting NRF2. Ann. Hematol..

[184] Chakraborty I., Mishra R., Gachhui R., Kar M. (2012). Distortion of β-Globin Chain of Hemoglobin Alters the Pathway of Erythrocytic Glucose Metabolism through Band 3 Protein. Arch. Med. Res..

[185] Ting Y.L.T., Naccarato S., Qualtieri A., Chidichimo G., Brancati C. (1994). In Vivo Metabolic Studies of Glucose, ATP and 2, 3-DPG in Β-thalassaemia Intermedia, Heterozygous Β-thalassaemic and Normal Erythrocytes: 13C and 31P MRS Studies. Br. J. Haematol..

[186] Rab M.A.E., van Oirschot B.A., van Straaten S., Biemond B.J., Bos J., Kosinski P.A., Kung C., van Beers E.J., van Wijk R. (2019). Decreased Activity and Stability of Pyruvate Kinase in Hereditary Hemolytic Anemia: A Potential Target for Therapy by AG-348 (Mitapivat), an Allosteric Activator of Red Blood Cell Pyruvate Kinase. Blood.

[187] Kuo K.H.M. (2023). Pyruvate Kinase Activators: Targeting Red Cell Metabolism in Thalassemia. Hematology.

[188] Taher A.T., Al-Samkari H., Aydinok Y., Besser M., Boscoe A.N., Dahlin J.L., De Luna G., Estepp J.H., Gheuens S., Gilroy K.S. (2025). Mitapivat in Adults with Non-Transfusion-Dependent α-Thalassaemia or β-Thalassaemia (ENERGIZE): A Phase 3, International, Randomised, Double-Blind, Placebo-Controlled Trial. Lancet.

[189] van Dijk M.J., Ruiter T.J.J., van der Veen S., Rab M.A.E., van Oirschot B.A., Bos J., Derichs C., Rijneveld A.W., Cnossen M.H., Nur E. (2024). Metabolic Blood Profile and Response to Treatment with the Pyruvate Kinase Activator Mitapivat in Patients with Sickle Cell Disease. HemaSphere.

[190] Miwa S., Kashyap S., Chini E., von Zglinicki T. (2022). Mitochondrial Dysfunction in Cell Senescence and Aging. J. Clin. Invest..

[191] Lyu J., Ni M., Weiss M.J., Xu J. (2024). Metabolic Regulation of Erythrocyte Development and Disorders. Exp. Hematol..

[192] Khungwanmaythawee K., Sornjai W., Paemanee A., Jaratsittisin J., Fucharoen S., Svasti S., Lithanatudom P., Roytrakul S., Smith D.R. (2016). Mitochondrial Changes in Β0-Thalassemia/Hb E Disease. PLoS ONE.

[193] Leecharoenkiat A., Wannatung T., Lithanatudom P., Svasti S., Fucharoen S., Chokchaichamnankit D., Srisomsap C., Smith D.R. (2011). Increased Oxidative Metabolism Is Associated with Erythroid Precursor Expansion in Β0-Thalassaemia/Hb E Disease. Blood Cells Mol. Dis..

[194] Suriyun T., Winichagoon P., Fucharoen S., Sripichai O. (2022). Impaired Terminal Erythroid Maturation in Β0-Thalassemia/HbE Patients with Different Clinical Severity. J. Clin. Med..

[195] Suragani R.N.V.S., Cadena S.M., Cawley S.M., Sako D., Mitchell D., Li R., Davies M.V., Alexander M.J., Devine M., Loveday K.S. (2014). Transforming Growth Factor-β Superfamily Ligand Trap ACE-536 Corrects Anemia by Promoting Late-Stage Erythropoiesis. Nat. Med..

[196] Matte A., Wilson A.B., Gevi F., Federti E., Recchiuti A., Ferri G., Brunati A.M., Pagano M.A., Russo R., Leboeuf C. (2023). Mitapivat Reprograms the RBC Metabolome and Improves Anemia in a Mouse Model of Hereditary Spherocytosis. JCI Insight.

[197] Sun Y., Benmhammed H., Al Abdullatif S., Habara A., Fu E., Brady J., Williams C., Ilinski A., Sharma A., Mahdaviani K. (2024). PGC-1α Agonism Induces Fetal Hemoglobin and Exerts Antisickling Effects in Sickle Cell Disease. Sci. Adv..

[198] Di Pierro E., Di Stefano V., Migone De Amicis M., Graziadei G. (2025). Are Mitochondria a Potential Target for Treating β-Thalassemia?. J. Clin. Med..

[199] Jabeen S., Riaz R., Hospital K., Khan R., Zhaira D., Rafaqat S., Fatima M., Ali S. (2025). Hepcidin Levels, Markers of Iron Overload, and Liver Damage in Patients with Beta Thalassemia Major. Rev. J. Neurol. Med. Sci. Rev..

[200] Ganz T., Nemeth E., Rivella S., Goldberg P., Dibble A.R., McCaleb M.L., Guo S., Monia B.P., Barrett T.D. (2023). TMPRSS6 as a Therapeutic Target for Disorders of Erythropoiesis and Iron Homeostasis. Adv. Ther..

[201] Katsarou A., Pantopoulos K. (2018). Hepcidin Therapeutics. Pharmaceuticals.

[202] Nyffenegger N., Flace A., Doucerain C., Dürrenberger F., Manolova V. (2021). The Oral Ferroportin Inhibitor VIT-2763 Improves Erythropoiesis without Interfering with Iron Chelation Therapy in a Mouse Model of β-Thalassemia. Int. J. Mol. Sci..

[203] Wallace D., Mcdonald C., Secondes E., Ostini L., Rishi G., Hooper J., Velasco G., Ramsay A., López-Otín C., Subramaniam V. (2013). An Essential Role For Transferrin Receptor 2 In Erythropoiesis During Iron Restriction. Blood.

[204] Longo F., Piga A. (2022). Does Hepcidin Tuning Have a Role among Emerging Treatments for Thalassemia?. J. Clin. Med..

[205] Chatzikalil E., Stergiou I.E., Papadakos S.P., Konstantinidis I., Theocharis S. (2024). The Clinical Relevance of the EPH/Ephrin Signaling Pathway in Pediatric Solid and Hematologic Malignancies. Int. J. Mol. Sci..

[206] Arvanitakis K., Papadakos S.P., Vakadaris G., Chatzikalil E., Stergiou I.E., Kalopitas G., Theocharis S., Germanidis G. (2024). Shedding Light on the Role of LAG-3 in Hepatocellular Carcinoma: Unraveling Immunomodulatory Pathways. Hepatoma Res..

[207] Papadakos S.P., Chatzikalil E., Vakadaris G., Reppas L., Arvanitakis K., Koufakis T., Siakavellas S.I., Manolakopoulos S., Germanidis G., Theocharis S. (2024). Exploring the Role of GITR/GITRL Signaling: From Liver Disease to Hepatocellular Carcinoma. Cancers.

[208] Clemente-Suárez V.J., Martín-Rodríguez A., Redondo-Flórez L., López-Mora C., Yáñez-Sepúlveda R., Tornero-Aguilera J.F. (2023). New Insights and Potential Therapeutic Interventions in Metabolic Diseases. Int. J. Mol. Sci..

[209] Papadakos S.P., Chatzikalil E., Arvanitakis K., Vakadaris G., Stergiou I.E., Koutsompina M.-L., Argyrou A., Lekakis V., Konstantinidis I., Germanidis G. (2024). Understanding the Role of Connexins in Hepatocellular Carcinoma: Molecular and Prognostic Implications. Cancers.

[210] Zhou H., Xiang W., Zhou G., Rodrigues-Lima F., Guidez F., Wang L. (2024). Metabolic Dysregulation in Myelodysplastic Neoplasm: Impact on Pathogenesis and Potential Therapeutic Targets. Med. Oncol..

[211] Gavriatopoulou M., Paschou S.A., Ntanasis-Stathopoulos I., Dimopoulos M.A. (2021). Metabolic Disorders in Multiple Myeloma. Int. J. Mol. Sci..

[212] Mokhtari R.B., Homayouni T.S., Baluch N., Morgatskaya E., Kumar S., Das B., Yeger H. (2017). Combination Therapy in Combating Cancer. Oncotarget.

[213] Lehár J., Krueger A.S., Avery W., Heilbut A.M., Johansen L.M., Price E.R., Rickles R.J., Short Iii G.F., Staunton J.E., Jin X. (2009). Synergistic Drug Combinations Tend to Improve Therapeutically Relevant Selectivity. Nat. Biotechnol..

[214] Zhou Y., Tao L., Qiu J., Xu J., Yang X., Zhang Y., Tian X., Guan X., Cen X., Zhao Y. (2024). Tumor Biomarkers for Diagnosis, Prognosis and Targeted Therapy. Signal Transduct. Target. Ther..

[215] O’Brien III W.G., Berka V., Tsai A.-L., Zhao Z., Lee C.C. (2015). CD73 and AMPD3 Deficiency Enhance Metabolic Performance via Erythrocyte ATP That Decreases Hemoglobin Oxygen Affinity. Sci. Rep..

[216] Jaafar L., Kourie C., El-Mallah C., Obeid O. (2025). 2,3-Diphosphoglycerate: The Forgotten Metabolic Regulator of Oxygen Affinity. Br. J. Nutr..

[217] Koehl B., Claude L., Reminy K., Tarer V., Baccini V., Romana M., Colin-Aronovicz Y., Damaraju V.L., Sawyer M., Peyrard T. (2023). Erythrocyte Type 1 Equilibrative Nucleoside Transporter Expression in Sickle Cell Disease and Sickle Cell Trait. Br. J. Haematol..

[218] Dalamaga M. (2024). Clinical Metabolomics: Useful Insights, Perspectives and Challenges. Metab. Open.

[219] Hertzog A., Selvanathan A., Devanapalli B., Ho G., Bhattacharya K., Tolun A. (2021). A Narrative Review of Metabolomics in the Era of “-Omics”: Integration into Clinical Practice for Inborn Errors of Metabolism. Transl. Pediatr..

[220] Armakolas A., Kotsari M., Koskinas J. (2023). Liquid Biopsies, Novel Approaches and Future Directions. Cancers.

[221] Ali H. (2023). Artificial Intelligence in Multi-Omics Data Integration: Advancing Precision Medicine, Biomarker Discovery and Genomic-Driven Disease Interventions. Int. J. Sci. Res. Arch..

[222] Goetz L.H., Schork N.J. (2018). Personalized Medicine: Motivation, Challenges, and Progress. Fertil. Steril..

